# RUNX2 stabilization by long non-coding RNAs contributes to hypertrophic changes in human chondrocytes

**DOI:** 10.7150/ijbs.74895

**Published:** 2023-01-01

**Authors:** Dong Suk Yoon, Eun-Ji Kim, Sehee Cho, Soyeong Jung, Kyoung-Mi Lee, Kwang Hwan Park, Jin Woo Lee, Sung-Hwan Kim

**Affiliations:** 1 Department of Orthopaedic Surgery, Yonsei University College of Medicine, Seoul 03722, South Korea.; 2 Brain Korea 21 PLUS Project for Medical Science, Yonsei University College of Medicine, Seoul 03722, South Korea.; 3 Severance Biomedical Science Institute, Yonsei University College of Medicine, Seoul 03722, South Korea.; 4 Arthroscopy and Joint Research Institute, Yonsei University College of Medicine, Seoul 03722, South Korea.; 5 Department of Orthopedic Surgery, Gangnam Severance Hospital, Yonsei University College of Medicine, Seoul 06273, South Korea.

**Keywords:** Chondrocyte hypertrophy, mesenchymal stem cells, RUNX2, long non-coding RNAs, osteoarthritis

## Abstract

**Background:** Chondrocyte hypertrophy has been implicated in endochondral ossification and osteoarthritis (OA). In OA, hypertrophic chondrocytes contribute to the destruction and focal calcification of the joint cartilage. Although studies in this field have remarkably developed the modulation of joint inflammation using gene therapy and regeneration of damaged articular cartilage using cell therapy, studies that can modulate or prevent hypertrophic changes in articular chondrocytes are still lacking.

**Methods:** *In vitro* hypertrophic differentiation and inflammation assays were conducted using human normal chondrocyte cell lines, TC28a2 cells. Human cartilage tissues and primary articular chondrocytes were obtained from OA patients undergoing total knee arthroplasty. Long non-coding RNAs (lncRNAs), LINC02035 and LOC100130207, were selected through RNA-sequencing analysis using RNAs extracted from TC28a2 cells cultured in hypertrophic medium. The regulatory mechanism was evaluated using western blotting, real-time quantitative polymerase chain reaction, osteocalcin reporter assay, RNA-immunoprecipitation (RNA-IP), RNA-*in situ* hybridization, and IP.

**Results:** LncRNAs are crucial regulators of various biological processes. In this study, we identified two important lncRNAs, LINC02035 and LOC100130207, which play important roles in hypertrophic changes in normal chondrocytes, through RNA sequencing. Interestingly, the expression level of RUNX2, a master regulator of chondrocyte hypertrophy, was regulated at the post-translational level during hypertrophic differentiation of the normal human chondrocyte cell line, TC28a2. RNA-immunoprecipitation proved the potential interaction between RUNX2 protein and both lncRNAs. Knockdown (KD) of LINC02035 or LOC100130207 promoted ubiquitin-mediated proteasomal degradation of RUNX2 and prevented hypertrophic differentiation of normal chondrocyte cell lines, whereas overexpression of both lncRNAs stabilized RUNX2 protein and generated hypertrophic changes. Furthermore, the KD of the two lncRNAs mitigated the destruction of important cartilage matrix proteins, COL2A1 and ACAN, by hypertrophic differentiation or inflammatory conditions. We also confirmed that the phenotypic changes raised by the two lncRNAs could be rescued by modulating *RUNX2* expression. In addition, the KD of these two lncRNAs suppressed hypertrophic changes during chondrogenic differentiation of mesenchymal stem cells.

**Conclusion:** Therefore, this study suggests that LINC02035 and LOC100130207 contribute to hypertrophic changes in normal chondrocytes by regulating RUNX2, suggesting that these two novel lncRNAs could be potential therapeutic targets for delaying or preventing OA development, especially for preventing chondrocyte hypertrophy.

## Introduction

Osteoarthritis (OA) is characterized by substantial loss of articular cartilage due to multiple changes that occur within the tissue, such as dysfunction of chondrocytes and inflammation of synovial tissue 1, 2. Articular cartilage is largely divided into two layers, calcified and non-calcified zones, which can be divided by the tidemark. The non-calcified zone in the articular cartilage includes a superficial region that can determine the dynamic properties of tissue homeostasis 3. In general, chondrocytes present in non-calcified regions release cartilage matrix proteins, such as COL2A1 and ACGN, whereas chondrocytes residing in the calcified zone display a hypertrophic morphology distinguishable in the expression of RUNX2 and COL10A1^4^. However, chondrocytes in non-calcified regions can also be positive for hypertrophic markers such as RUNX2, COL10A1, and MMP13 in the osteoarthritic environment, suggesting that hypertrophic changes in chondrocytes residing in non-calcified regions are largely involved in OA progression 5. Therefore, hypertrophic differentiation of chondrocytes within the superficial region of the articular cartilage could be one of the major phenotypic changes in OA development.

Multiple factors such as transcription factors, cytokines, and matrix proteins are involved in chondrocyte hypertrophy 6. RUNX2 is a major regulator of chondrocyte hypertrophy and bone metabolism 7, and RUNX2 overexpression induces hypertrophic differentiation of non-hypertrophic chondrocytes 8. Recent studies have emphasized the importance of RUNX2 in OA development and chondrocyte hypertrophy 9. Cartilage-specific expression of Runx2 exacerbates post-traumatic OA development in mice 10, whereas *Runx2* conditional knockout (cKO) in chondrocytes delays OA progression in mice with destabilization of the medial meniscus (DMM) during surgery 11, suggesting that Runx2 is deeply involved in OA pathogenesis. A previous study has shown that RUNX2 binds to the proximal promoter of COL10A1 to activate its transcription 12. This study proved that COL10A1 is a direct target of RUNX2 during chondrogenesis and has multiple RUNX2 binding sites within the proximal promoters of humans, mice, and chicks. Since COL10A1 is a specific marker of chondrocyte hypertrophy 13, 14 it has been shown that the regulation of COL10A1 expression by RUNX2 is a key regulatory axis of chondrocyte hypertrophy. Although the importance of RUNX2 in chondrocyte hypertrophy has been elucidated, studies on the regulation of RUNX2 activity during chondrocyte hypertrophy are lacking. RUNX2 can be regulated at all transcriptional, post-transcriptional, and post-translational stages in the central dogma 15, 16. Therefore, to develop biological techniques that can control chondrocyte hypertrophy by targeting RUNX2, it is necessary to conduct a multifaceted review and study the regulation of RUNX2 during chondrocyte hypertrophy.

In this study, we found that RUNX2 was regulated at the post-translational level during hypertrophic differentiation of the chondrocyte cell line TC28a2, and many long-non-coding RNAs (lncRNAs) were upregulated during this process. We also identified two novel RUNX2-binding lncRNAs that were shown to be involved in hypertrophic changes in chondrocytes. Both lncRNAs are not only required for stabilization of RUNX2 protein but are also involved in hypertrophic changes during chondrogenic differentiation of mesenchymal stem cells (MSCs). These findings can help us assess the different mechanisms of chondrocyte hypertrophy and allow us to identify potential therapeutic targets of lncRNAs and prevent hypertrophic changes in non-hypertrophic chondrocytes.

## Results

### Upregulation of RUNX2 is essential for hypertrophic changes and inflammatory responses of chondrocytes

**Figure 1:**
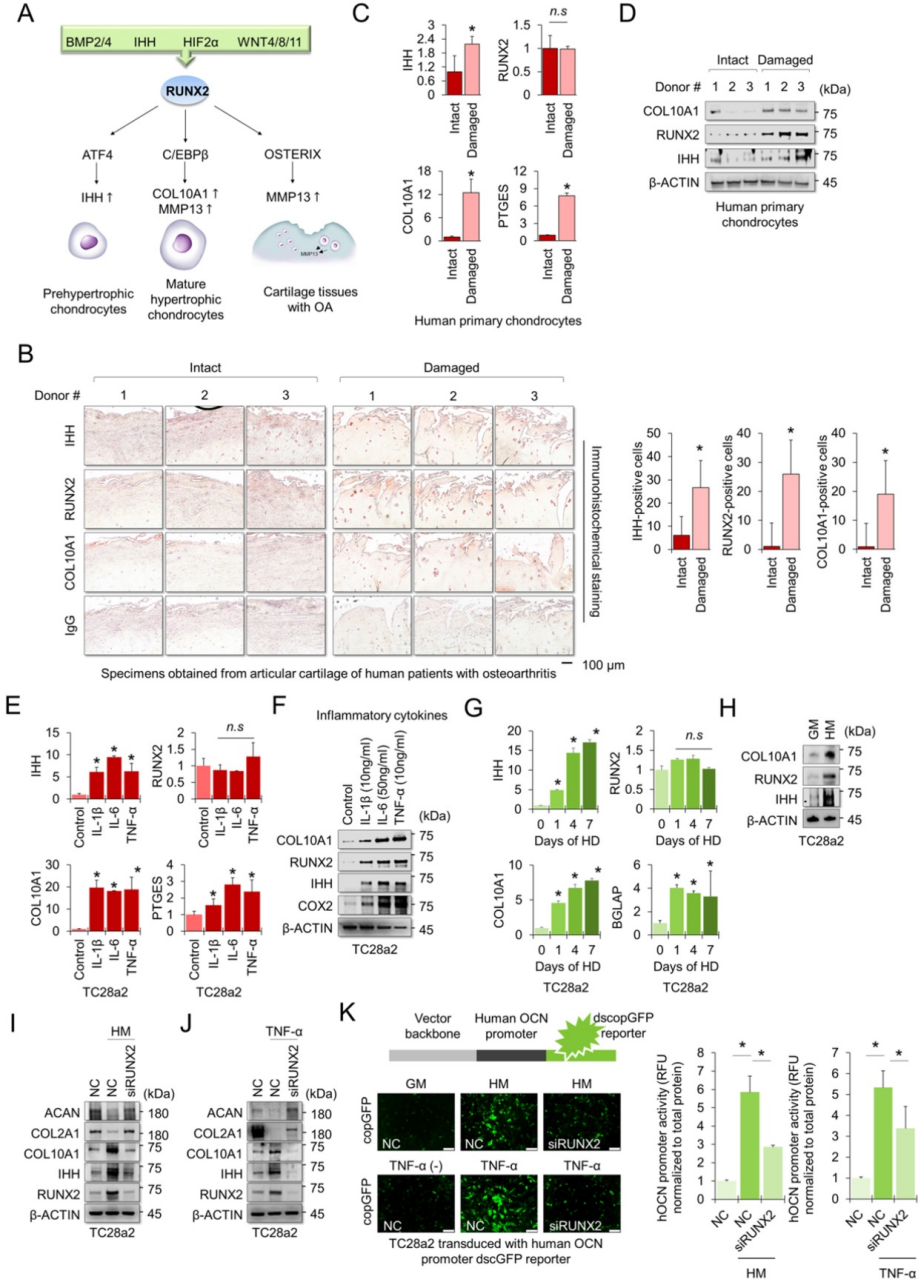
** RUNX2 is a key mediator of chondrocyte hypertrophy. (A)** Schematic diagram of the role of RUNX2 in the process of chondrocyte hypertrophy and osteoarthritis (OA). Activation of RUNX2 by various stimuli induces hypertrophy of articular chondrocytes, inducing the expression of downstream genes, thereby accelerating OA progression. **(B)** Representative images of immunostaining in human cartilage isolated from knee OA patients undergoing total knee replacement. Scale bars, 100 µm for immunostaining. IHH-, RUNX2-, and COL10A1-positive cells were counted from the total cell population per field in immunohistochemical sections. *P < 0.05 in comparison with intact groups (n = 6 experimental replicates). **(C)** qRT-PCR analysis of *IHH*, *RUNX2*, *COL10A1*, and *PTGES* mRNA expressions in primary chondrocytes isolated from knee OA patients undergoing total knee replacement. *P < 0.05 in comparison with control group (n = 6 experimental replicates). n.s., not significant. **(D)** Representative images of western blot analysis for protein levels of IHH, RUNX2, and COL10A1 in primary chondrocytes isolated from knee OA patients undergoing total knee replacement. **(E)** qRT-PCR analysis of *IHH*, *RUNX2*, *COL10A1*, and *PTGES* mRNA expression in TC28a2 cells that were treated with or without inflammatory cytokines (10 ng/ml IL-1β, 50 ng/ml IL-6, or 10 ng/ml TNF-α). *P < 0.05 in comparison with control group (n = 3 experimental replicates). n.s., not significant. **(F)** Representative images of western blot analysis for protein levels of COX2, IHH, RUNX2, and COL10A1 in TC28a2 cells that were treated with or without inflammatory cytokines (10 ng/ml IL-1β, 50 ng/ml IL-6, or 10 ng/ml TNF-α). β-ACTIN was used as a loading control. **(G)** qRT-PCR analysis of *IHH*, *RUNX2*, *COL10A1*, and *BGLAP* mRNA expression in TC28a2 cells that were treated with or without hypertrophic medium. *P < 0.05 in comparison with control group (n = 3 experimental replicates). **(H)** Representative images of western blot analysis for protein levels of COX2, IHH, RUNX2, and COL10A1 in TC28a2 cells that were treated with or without hypertrophic medium. β-ACTIN was used as a loading control. GM, growth medium; HM, hypertrophic medium. **(I, J)** Representative images of western blot analysis for protein levels of RUNX2, IHH, COL10A1, COL2A1, and ACAN in siRNA-transfected TC28a2 cells that were treated with or without hypertrophic medium/TNF-α (10 ng/ml). β-ACTIN was used as a loading control. NC, non-targeting control siRNA; siRUNX2, siRNA targeting *RUNX2*. **(K)** Fluorescence images showing the activity of OCN promoter and the OCN promoter reporter quantity. TC28a2 cells were transduced with lentivirus of OCN pGreenZeo differentiation reporter. Bar graph indicates the fluorescence-based activities of OCN promoter. *P < 0.05 compared with the NC group (n = 3 experimental replicates). OCN, osteocalcin; dscopGFP, destabilized form of copepod green fluorescence protein. Scale bars, 100 µm.

The RUNX2 transcription factor is a central regulator of hypertrophic changes in chondrocytes and is involved in the degradation of the cartilage matrix 17 (Figure 1A). To confirm the regulatory role of RUNX2 in hypertrophic changes in chondrocytes and degradation of cartilage matrix, we first performed immunohistochemistry (IHC) to detect hypertrophic markers, including RUNX2, in cartilage tissues obtained from patients with OA. The chondrocyte hypertrophic markers IHH, RUNX2, and COL10A1 were highly detected in damaged cartilage tissue, whereas little was detected in intact cartilage tissue (Figure 1B). The mRNA levels of *IHH*, *COL10A1*, and *PTGES* were highly expressed in primary chondrocytes isolated from damaged cartilage tissue (Figure 1C), and the protein levels of hypertrophy markers, including RUNX2, were also highly detected in the cells (Figure 1D). To observe changes in chondrocyte hypertrophy in the *in vitro* environment, TC28a2 cells, a normal chondrocyte cell line, were employed and the inflammatory cytokines interleukin-1β (IL-1β), interleukin-6 (IL-6) and tumor necrosis factor-α (TNF-α) were used to mimic the *in vitro* osteoarthritic milieu. We confirmed that inflammatory cytokines upregulated the mRNA and protein levels of PTGES (an inflammation marker COX2) and the hypertrophic markers IHH and COL10A1 (Figure 1E-F). Interestingly, the amount of RUNX2 protein was upregulated by inflammatory cytokines or in damaged cartilage tissues (Figure 1D and 1F), but the mRNA levels did not change (Figure 1C and 1E). Hypertrophy medium 18 was then used to create an *in vitro* environment specific for chondrocyte hypertrophy. Likewise, the hypertrophic medium upregulated both mRNA and protein levels of IHH and COL10A1, markers of chondrocyte hypertrophy in TC28a2 cells, whereas the hypertrophic medium improved the protein level of RUNX2 but did not change the mRNA level (Figure 1G-H). To clarify whether RUNX2 is functionally associated with hypertrophic changes in chondrocytes, we performed siRNA-mediated knockdown (KD) experiments in hypertrophic and inflammatory environments. The results showed that KD of *RUNX2* protected chondrocytes from hypertrophic medium- or TNF-α treatment-mediated loss of the cartilage matrix proteins COL2A1 and ACAN. In addition, the KD of *RUNX2* prevented the upregulation of IHH and COL10A1 (Figure 1I-J). Osteocalcin (OCN) is the most abundant protein produced by osteoblasts 19 in the bone and is involved in matrix calcification in cartilage tissues 20. To investigate whether OCN activity in chondrocytes was regulated by hypertrophic medium or TNF-α treatment, we performed a human OCN pGreenZeo (copGFP) differentiation reporter assay. TC28a2 cells were transduced with the lentiviral vector encoding the OCN promoter and copGFP and then used for the reporter assay after selection with zeomycin. As a result, a strong copGFP signal was detected in TC28a2 cells treated with hyperophic medium or TNF-α, and it was shown that the increased copGFP signal was greatly reduced by siRNA-mediated KD of *RUNX2* (Figure 1K). Therefore, we conclude that RUNX2 is a master regulator of hypertrophic changes in chondrocytes.

### LncRNAs interact with RUNX2 protein in chondrocytes

**Figure 2:**
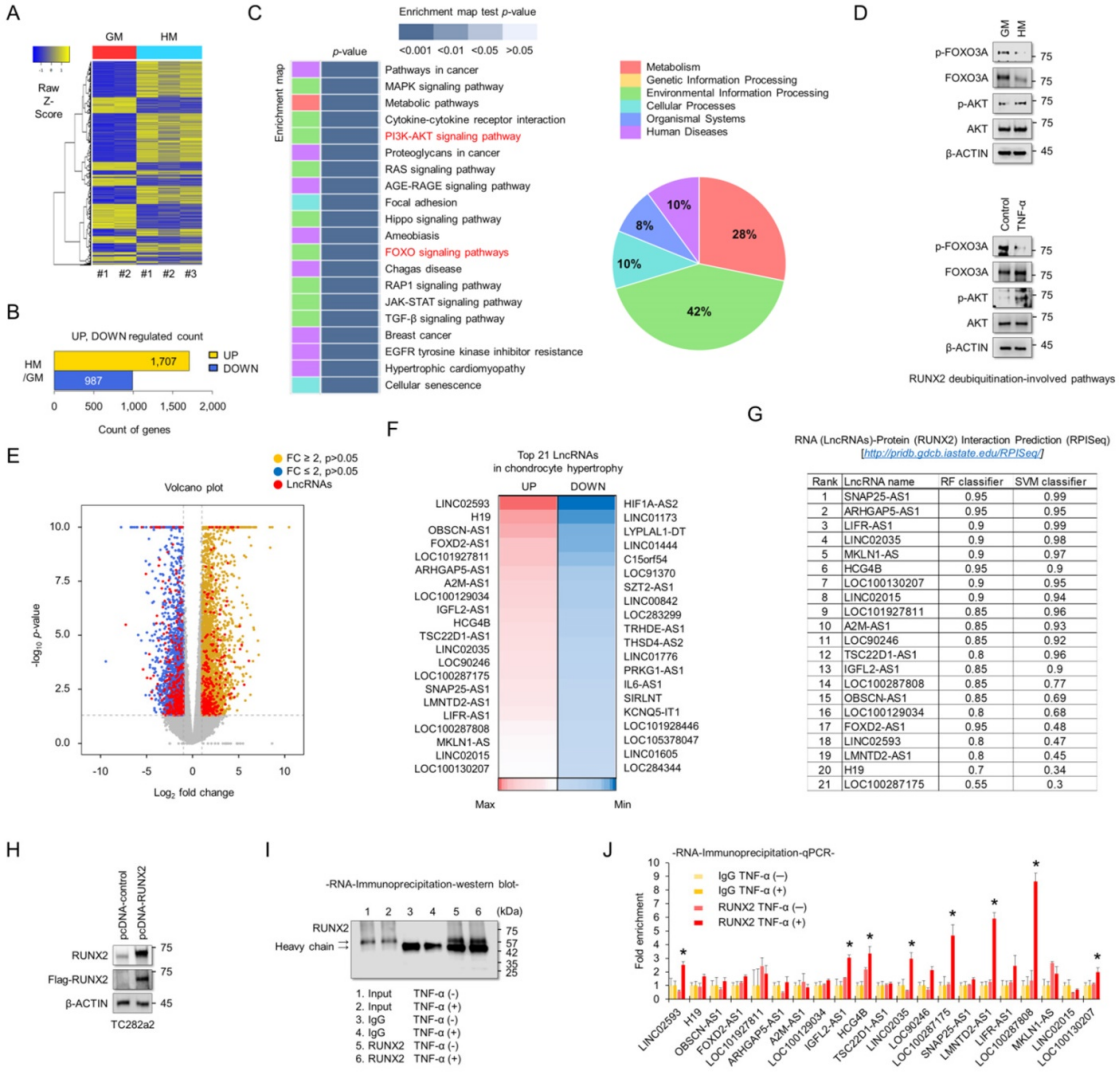
** Screening of lncRNAs exhibiting different expression between normal and hypertrophic chondrocytes, and identification of their potential interactions with RUNX2. (A)** Hierarchical clustering of RNA-sequencing data. A heatmap was generated from RNA-sequencing data using RNA extracted from TC28a2 cells grown with growth medium (GM) or hypertrophic medium (HM). The results were compared between GM (n = 2) and HM (n = 3). The relative expression of total mRNAs is depicted according to the color scale shown on the left. Yellow represents relatively high expression and blue means relatively low expression, and -1, 0, 1 are fold changes in the corresponding spectrum. **(B)** A bar graph shows numbers of up-regulated (yellow) and down-regulated (blue) genes. **(C)** Kegg pathway annotation categories for target gene functions and a diagram for the related cellular pathways. **(D)** Representative images of western blot analysis for protein levels of AKT, p-AKT, FOXO3A, and p-FOXO3A in TC28a2 cells that were treated with or without hypertrophic medium/TNF-α. β-ACTIN was used as a loading control. GM, growth medium; HM, hypertrophic medium. **(E)** Volcano plots of differentially expressed RNAs between GM and HM groups. Red dots indicate differentially expressed lncRNAs between two groups. Volcano plot of all genes expressed greater than 1 count-per-million where the observed log2 fold change is on the x-axis and the unadjusted P value converted to the -log10 scale is on the y-axis. **(F)** A heatmap of differentially expressed lncRNAs during hypertrophic differentiation of TC28a2 cells. The data shows the top 21 lncRNAs which have shown a distinctively increased expression pattern (Red; > fold change 4) or with the greatest decrease (Blue) in expression levels during hypertrophic differentiation. **(G)** RPISeq analysis was used to predict the interaction probability of selected lncRNAs with RUNX2. An RF and SVM > 0.5 was considered to have the ability of binding (*http://pridb.gdcb.iastate.edu/RPISeq/about.php#basic*). **(H)** Representative images of western blot analysis for protein levels of FLAG and RUNX2 in TC28a2 cells that were transfected with pcDNA-mock or pcDNA-RUNX2-FLAG plasmid vector. β-ACTIN was used as a loading control. **(I)** A representative image of western blot analysis for protein levels of RUNX2 using RNA-IP samples obtained from TC28a2 cells transfected with pcDNA-RUNX2 vector. RNA-IP was conducted by dividing the TNF-α-treated group and the non-TNF-α group here. **(J)** The bar graph displays the RNA-IP-qPCR fold enrichment of the indicated lncRNAs relative to the IgG control. RNA-IP was performed in TC28a2 cells transfected with the pcDNA-RUNX2 vector. *P < 0.05 in a comparison with the IgG control (n = 3 experimental replicates).

To understand the molecular mechanisms associated with chondrocyte hypertrophy, RNA sequencing analyses were performed using RNAs extracted from TC28a2 cells grown in hypertrophic medium. A heatmap was used to display the different transcripts between TC28a2 cells grown in either growth or hypertrophic medium (Figure 2A). A total of 1707 transcripts were upregulated (log2 [fold-change] > 2, FDR < 0.05) and 987 transcripts were downregulated (log2 [fold-change] < -2, FDR < 0.05) in TC28a2 cells grown in hypertrophic differentiation medium for 5 days (Figure 2B). KEGG pathway analysis showed changes in differentially regulated cell signaling pathways, with pathways related to environmental information processing and metabolism accounting for a significant proportion (Figure 2C). Among KEGG pathway enrichment analyses, we focused on changes in the PI3K-AKT and FOXO signaling pathways because these two signaling pathways are involved in the stability of the RUNX2 protein 21, 22. We found that AKT phosphorylation was increased in TC28a2 cells treated with hypertrophic medium or TNF-α, whereas FOXO3A phosphorylation was reduced (Figure 2D). These results imply that RUNX2 can be regulated at the post-translational level during hypertrophic differentiation of chondrocytes. The most significantly up- or down-regulated mRNAs closely associated with chondrocyte hypertrophy during OA progression are summarized in Table S1 and S2. In particular, well-known chondrocyte hypertrophy signaling pathways such as BMP and IHH were upregulated (Table S1). To confirm the accuracy of the RNA sequencing data, qPCR analysis was performed using cDNA samples reverse transcribed from RNAs extracted from TC28a2 cells grown in growth medium or hypertrophic medium. The qPCR results confirmed that the previously performed RNA sequencing data were relatively well reproduced (Figure S1). Interestingly, our RNA-sequencing analysis screened 2181 protein-coding genes and 223 lncRNAs among the differentially expressed transcripts. Volcano plots showed variations in the expression of lncRNAs and protein-coding mRNAs between the control and hypertrophic groups (Figure 2E). Detailed information on lncRNAs, including name, fold changes, and *p*-value, is summarized in Table S3 and S4. LcnRNAs are known to regulate the activity of their target proteins by physically binding to proteins, DNA or RNA^23^, ^24^. Since RUNX2 was shown to be regulated at the post-translational level in our experimental model and changes in many lncRNAs were observed in the RNA sequencing results, we wanted to determine which lncRNAs are involved in the stabilization of the RUNX2 protein. A heatmap showed 21 differentially expressed lncRNAs that were either upregulated by more than 4-fold or showed the greatest decrease in expression levels during hypertrophic differentiation (Figure 2F). We then performed a computer algorithm (RPISeq; RNA-Protein Interaction Prediction) to predict the possible lncRNAs that could interact with RUNX2 among the upregulated lncRNAs. If a value ≥ 0.5 or higher is recorded, the binding potential between the analyzed RNA and protein can be analyzed as positive (*http://pridb.gdcb.iastate.edu/RPISeq/about.php#basic*). However, most of the lncRNAs analyzed were positive (Figure 2G). We thus performed RNA-immunoprecipitation (RNA-IP) to identify lncRNAs capable of binding to RUNX2. First, the vector encoding RUNX2 was transfected into TC28a2 cells (Figure 2H), and the IP for RUNX2 was confirmed (Figure 2I). As a result, a total of 8 lncRNAs were enriched at RUNX2 protein during the inflammatory response in TC28a2 cells (Figure 2J).

### LINC02035 and LOC100130207 are novel lncRNAs that interact with RUNX2 in chondrocytes

**Figure 3:**
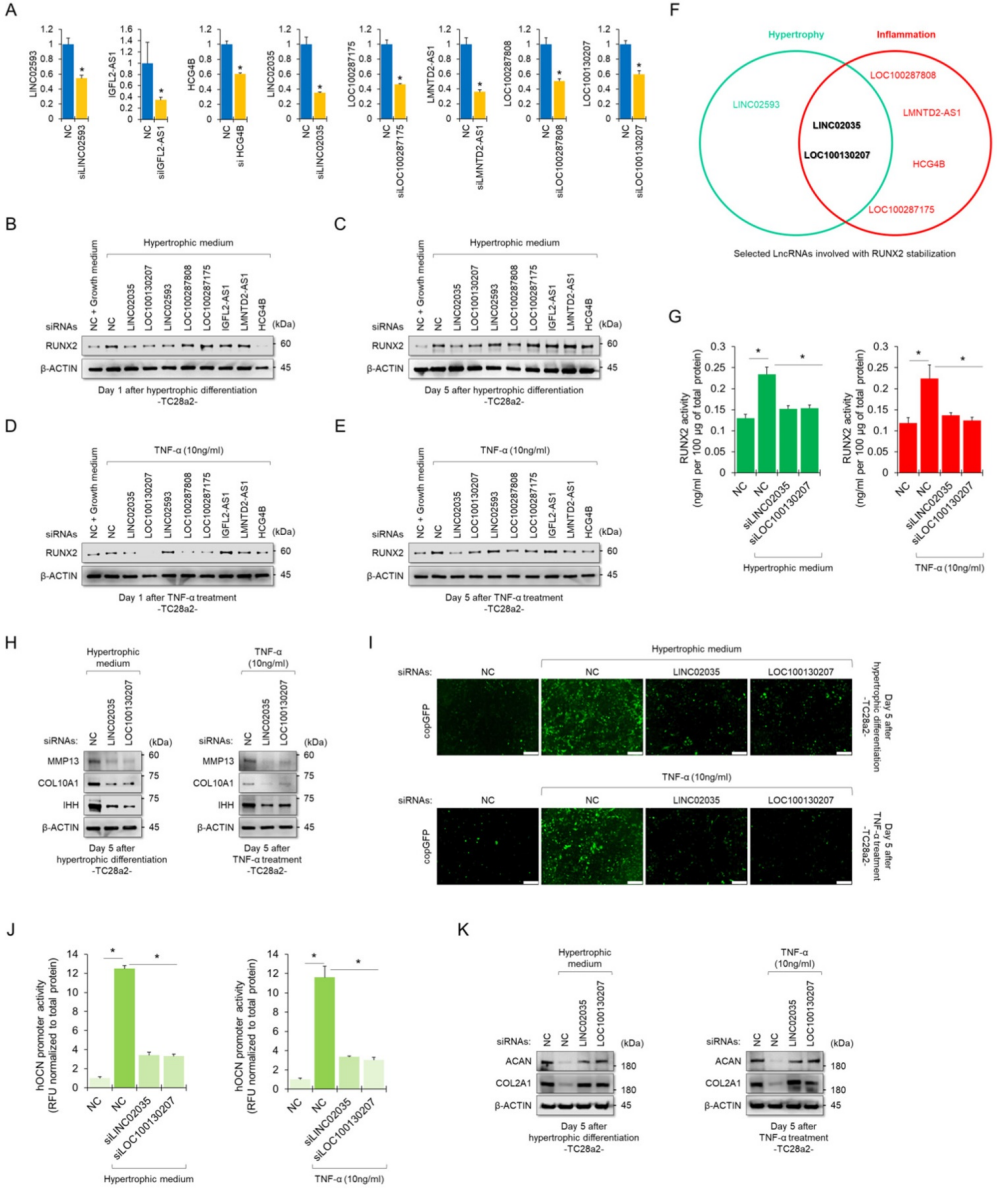
** Upregulation of LINC02035 and LOC100130207 is implicated with hypertrophic changes in normal chondrocytes. (A)** qRT-PCR analysis of each lncRNA expression in TC28a2 cells transfected with siRNAs (100 nM) targeting each lncRNA. The qPCR analysis was performed to confirm the efficiency of each siRNA used in this study for lncRNA knockdown. *P < 0.05 in comparison with NC group (n = 3 experimental replicates). NC, non-targeting control siRNA. **(B-E)** Representative images of western blot analysis for protein levels of RUNX2 in each siRNA-transfected TC28a2 cells that were treated with or without hypertrophic medium/TNF-α (10 ng/ml). β-ACTIN was used as a loading control. Western blot analysis was performed on day 1, which is an early stage of hypertrophic differentiation or inflammatory response, and day 5, which is a late stage, respectively. **(F)** The diagram shows the intersection of groups that did not change the amount of RUNX2 protein in both the early and late stages despite the hypertrophic medium or TNF-α treatment in TC28a2 cells when each siRNA was transfected. **(G)** Effects of knockdown of LINC02035 or LOC100130207 on RUNX2 activity were determined by enzyme-linked immuno-sorbent assay (ELISA). Hypertrophic medium or TNF-α was treated in each siRNA-transfected TC28a2 cells, and the cells were lysed in order to obtain total protein. RUNX2 activities were normalized to 100 µg of total protein. *P < 0.05 in comparison with NC group (n = 3 experimental replicates). **(H)** Representative images of western blot analysis for protein levels of IHH, COL10A1, and MMP13 in siRNA-transfected TC28a2 cells that were treated with or without hypertrophic medium/TNF-α (10 ng/ml). β-ACTIN was used as a loading control. **(I)** Fluorescence images showing the activity of OCN promoter and the OCN promoter reporter quantity. Lentivirus of OCN pGreenZeo differentiation reporter-transduced TC28a2 cells were transfected with siRNAs targeting LINC02035 or LOC100130207, and the cells were then treated with hypertrophic medium or TNF-α (10 ng/ml). Scale bars, 200 µm.** (J)** The bar graph indicates the fluorescence-based activities of OCN promoter. *P < 0.05 compared with the NC group (n = 3 experimental replicates). **(K)** Representative images of western blot analysis for protein levels of COL2A1 and ACAN in siRNA-transfected TC28a2 cells that were treated with or without hypertrophic medium/TNF-α (10 ng/ml). β-ACTIN was used as a loading control.

To determine whether RUNX2-binding lncRNAs could functionally contribute to the stability of the RUNX2 protein, we performed siRNA-mediated KD experiments for each lncRNA. The KD efficiency of each siRNA employed in this study was confirmed using real-time quantitative polymerase chain reaction (qPCR) (Figure 3A). Western blot results showed quantitative changes in RUNX2 protein when each lncRNA was knocked down, while TC28a2 cells were treated with hypertrophic medium or TNF-α (Figure 3B-E). In particular, TC28a2 cells transfected with siRNA against LINC02035 or LOC100130207 did not show an increase in RUNX2 protein levels, even under hypertrophic or inflammatory conditions (Figure 3F). For further analysis, RUNX2 activity was measured in TC28a2 cells transfected with siRNA against LINC02035 or LOC100130207. The results showed that RUNX2 activity was significantly reduced by KD of these two lncRNAs (Figure 3G). Thus, we concluded that LINC02035 and LOC100130207 functionally interact with RUNX2 in chondrocytes. Next, we wanted to determine whether LINC02035 or LOC100130207 was involved in the increase in chondrocyte hypertrophy markers and degradation of cartilage matrix proteins. Western blotting results showed that the KD of these two lncRNAs prevented the upregulation of hypertrophy markers such as IHH, COLA10A1, and MMP13 under hypertrophic or inflammatory conditions (Figure 3H). In addition, the promoter activity of OCN was not increased under each condition by KD of these two lncRNAs (Figure 3I-J). Moreover, the KD of these two lncRNAs could prevent the reduction of the cartilage matrix proteins COL2A1 and ACAN, which are destroyed by chondrocyte hypertrophy and the inflammatory response (Figure 3K). Therefore, we concluded that KD of LINC02035 and LOC100130207 protected chondrocytes from hypertrophy and inflammatory responses.

### LINC02035 and LOC100130207 are involved with stabilization of RUNX2 protein

**Figure 4:**
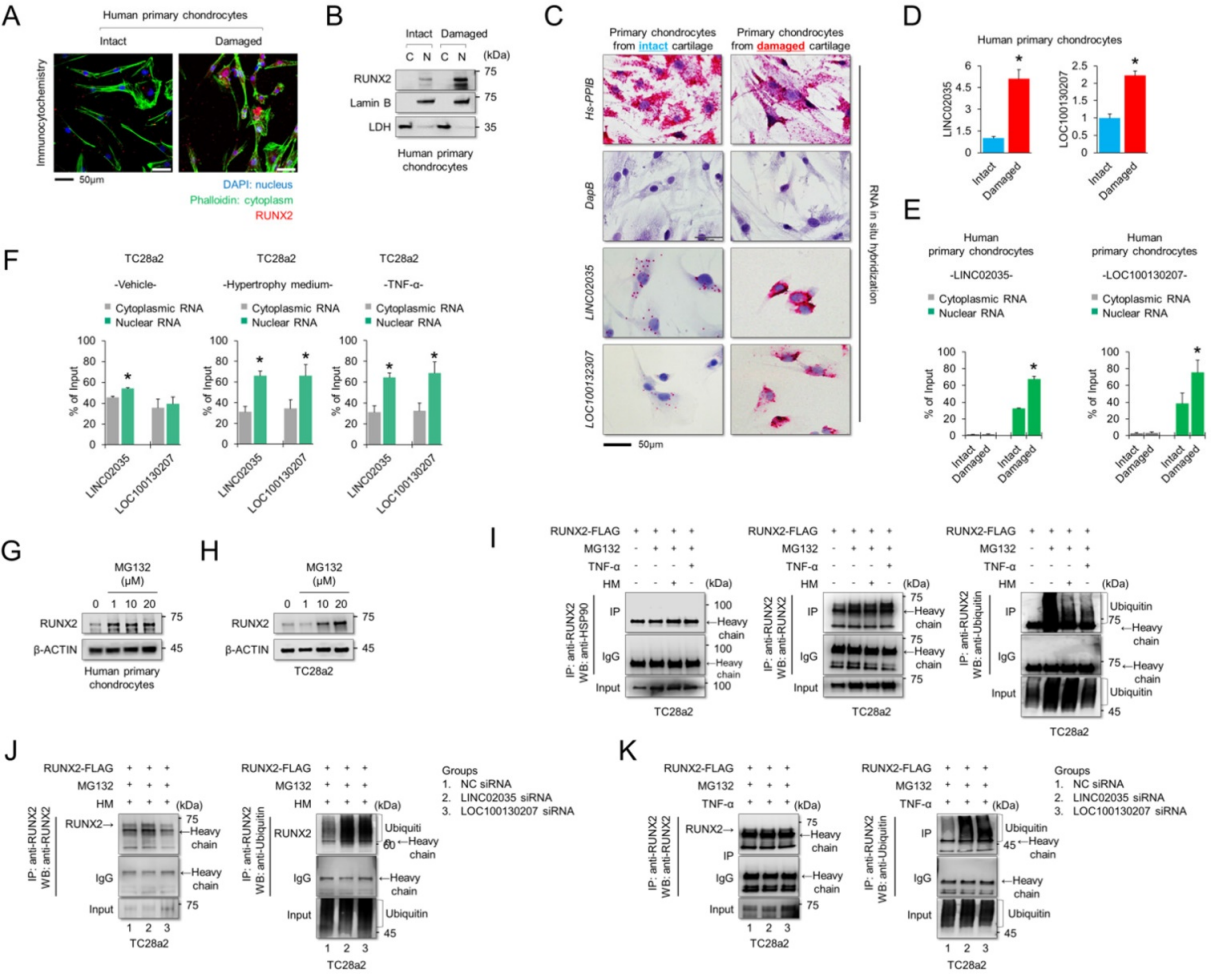
** LINC02035 and LOC100130207 are accumulated in chondrocytes isolated from damaged cartilage and involved with RUNX2 stabilization. (A)** Immunofluorescence was performed to observe the nuclear and cytosolic localization of RUNX2 in human primary chondrocytes isolated from intact or damaged cartilage tissues. The nuclei were stained with DAPI and cellular cytoskeleton was stained with Alexa Fluor 488 phalloidin. RUNX2 was stained with phycoerythrin (red)-conjugated secondary antibody. The images were obtained using confocal microscopy. Scale bar=50 µm. **(B)** The cells were fractionated into nuclear and cytosolic extracts, and the protein levels of RUNX2 were analyzed by western blot analysis. The protein level of LDH was used as a loading control for cytosolic extracts, and the protein level of LAMIN-B was used as a loading control for nuclear extracts. **(C)** Representative images of RNA *in situ* hybridization performed in primary chondrocytes isolated from intact or damaged cartilage tissues obtained from OA patients undergoing total knee replacement. Red dots indicate presence of each target RNA. Hs-PPIB, positive control; DapB, negative control; Scale bars, 50 µm. **(D)** qRT-PCR analysis of *LINC02035* and *LOC100130207* expressions in human primary chondrocytes isolated from knee OA patients undergoing total knee replacement. *P < 0.05 in comparison with intact group (n = 6 experimental replicates). **(E, F)** The distribution percentage of lncRNA LINC02035 and LOC100130207 in cytoplasm and nucleus of human primary chondrocytes and TC28a2 cells treated with hypertrophic medium or TNF-α (10 ng/ml). qPCR using subcellular fractionated RNAs was performed to explore the position changes of both lncRNAs. *P < 0.05 in comparison with cytoplasmic RNA group (n = 3 experimental replicates). **(G, H)** Representative images of western blot analysis for protein levels of RUNX2 in human primary chondrocytes or TC28a2 cells treated with or without MG132. β-ACTIN was used as a loading control. **(I)** IP/Western blot analysis was performed to investigate whether hypertrophic medium or TNF-α treatment affects ubiquitination of RUNX2 protein in TC28a2 cells. RUNX2 protein was immunoprecipitated using RUNX2 antibody followed western blot analysis using HSP90, RUNX2, and Ubiquitin antibodies to confirm the ubiquitination of RUNX2. **(J, K)** In the same way as above, IP/Western blot analysis was performed to investigate whether knockdown of LINC02035 or LOC100130207 prevents RUNX2 ubiquitination under hypertrophic or inflammatory conditions in TC28a2 cells treated with hypertrophic medium or TNF-α.

To understand the subcellular localization of RUNX2 and two lncRNAs, LINC02035 and LOC100130207, immunocytochemistry (for RUNX2 protein) and RNA *in situ* hybridization assays (for both lncRNAs) were performed using human primary chondrocytes isolated from knee cartilage of OA patients. We confirmed that the RUNX2 protein was strongly detected in the nucleus of chondrocytes isolated from damaged cartilage (Figure 4A). The fractionated western blot results also showed that the RUNX2 protein was detected only in the nucleus of chondrocytes isolated from damaged cartilage (Figure 4B). To further understand the molecular mechanisms of the lncRNAs LINC02035 and LOC100130207, we performed an *in situ* assay using primary chondrocytes derived from patients, because primary chondrocytes are a valuable model for studying OA pathogenesis. The results showed that both LINC02035 and LOC100130207 were highly expressed in chondrocytes isolated from the knee cartilage of patients with OA (Figure 4C). We then evaluated the mRNA levels and subcellular localization of both lncRNAs to elucidate their site of action using a commercially available kits in primary chondrocytes isolated from intact or damaged cartilage tissues. The results showed that both lncRNAs were more upregulated in primary chondrocytes isolated from damaged cartilage tissue than in intact cartilage tissue (Figure 4D). In primary chondrocytes, LINC02035 and LOC100130207 were merely detected in the cytoplasm of human primary chondrocytes and both lncRNAs were localized to nucleus (Figure 4E). Likewise, we also studied the subcellular localization of both lncRNAs in TC28a2 cells. The results showed that LINC02035 and LOC100130207 were evenly distributed in the nucleus and cytoplasm of normal chondrocytes (Figure 4F, left). However, both lncRNAs were preferentially localized to the nucleus and to a lesser extent to the cytoplasm after TC28a2 cells were treated with hypertrophic medium or TNF-α (Figure 4F, middle and right). To investigate whether RUNX2 is regulated at the post-translational level during hypertrophic changes in chondrocytes, human primary chondrocytes or TC28a2 cells were treated with the proteasome inhibitor MG132. The results showed that the protein level of RUNX2 was dose-dependently increased by MG132 treatment (Figure 4G-H), indicating that the upregulation of RUNX2 in osteoarthritic or hypertrophic chondrocytes occurs at the post-translational level. To examine whether both lncRNAs are involved in the stabilization of RUNX2 protein during hypertrophic differentiation and the inflammatory response in chondrocytes, IP was conducted to confirm the ubiquitination of RUNX2 protein. First, we investigated how the ubiquitination of RUNX2 protein is regulated during hypertrophic differentiation or the inflammatory response of chondrocytes, and as a result, it was confirmed that ubiquitination of RUNX2 was reduced by hypertrophic medium or TNF-α treatment (Figure 4I). However, ubiquitination of RUNX2 protein was clearly increased in TC28a2 cells transfected with siRNAs targeting LINC02035 or LOC100130207, even though the cells were treated with hypertrophic medium or TNF-α (Figure 4J-K). These results suggest that both lncRNAs upregulated during hypertrophic differentiation and the inflammatory response in chondrocytes contribute to the stabilization of the RUNX2 protein.

### Overexpression of LINC02035 and LOC100130207 generated hypertrophic changes in chondrocytes by stabilizing RUNX2 protein

**Figure 5:**
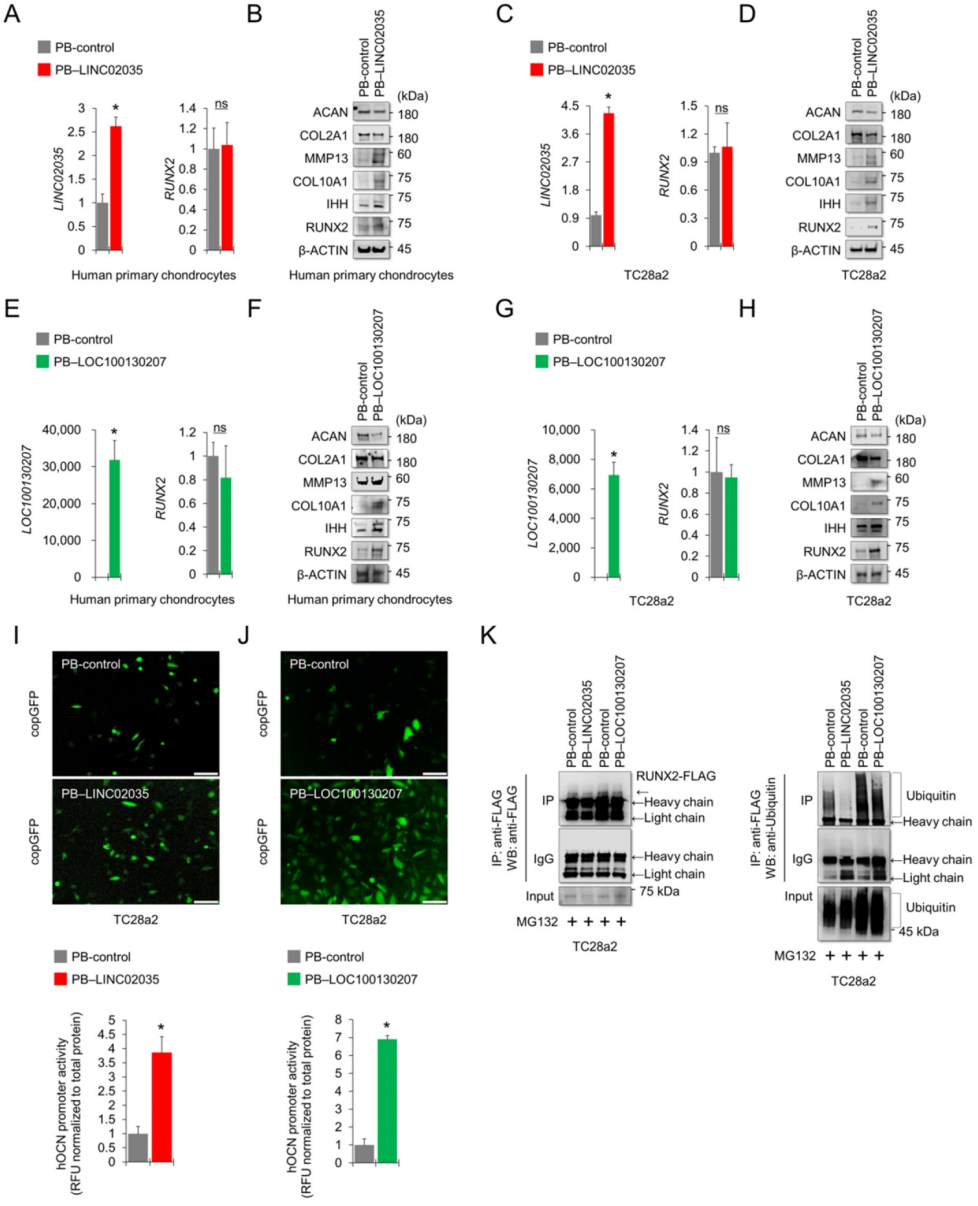
** Exogenously overexpressed LINC02035 or LOC100130207 induces hypertrophic changes in chondrocytes by stabilizing RUNX2 protein. (A)** qRT-PCR analysis of *LINC02035* and *RUNX2* mRNA expressions in human primary chondrocytes transfected with PB-control or PB-LINC02035 using electroporation with the Neon transfection system. Transfection efficiency was confirmed by qPCR analysis 48 h after electroporation. **(B)** Representative images of western blot analysis for protein levels of RUNX2, IHH, COL10A1, MMP13, COL2A1, and ACAN in human primary chondrocytes transfected with PB-control or PB-LINC02035 using electroporation. Western blot analysis was performed 72 h after electroporation. **(C)** qRT-PCR analysis of *LINC02035* and *RUNX2* mRNA expressions in TC28a2 cells transfected with PB-control or PB-LINC02035 using electroporation with the Neon transfection system. Transfection efficiency was confirmed by qPCR analysis 48 h after electroporation. **(D)** Representative images of western blot analysis for protein levels of RUNX2, IHH, COL10A1, MMP13, COL2A1, and ACAN in TC28a2 cells transfected with PB-control or PB-LINC02035 using electroporation. Western blot analysis was performed 72 h after electroporation. **(E)** qRT-PCR analysis of *LOC100130207* and *RUNX2* mRNA expressions in human primary chondrocytes transfected with PB-control or PB-LOC100130207 using a traditional cationic-lipid transfection reagent (Lipofectamine 2000). Transfection efficiency was confirmed by qPCR analysis 48 h after transfection. **(F)** Representative images of western blot analysis for protein levels of RUNX2, IHH, COL10A1, MMP13, COL2A1, and ACAN in human primary chondrocytes transfected with PB-control or PB-LOC100130207 using a traditional cationic-lipid transfection reagent. Western blot analysis was performed 72 h after transfection. **(G)** qRT-PCR analysis of *LOC100130207* and *RUNX2* mRNA expressions in TC28a2 cells transfected with PB-control or PB-LINC02035 using a traditional cationic-lipid transfection reagent. Transfection efficiency was confirmed by qPCR analysis 48 h after transfection. **(H)** Representative images of western blot analysis for protein levels of RUNX2, IHH, COL10A1, MMP13, COL2A1, and ACAN in TC28a2 cells transfected with PB-control or PB-LOC100130207 using a traditional cationic-lipid transfection reagent. Western blot analysis was performed 72 h after transfection. **(I, J)** Fluorescence images showing the activity of OCN promoter and the OCN promoter reporter quantity. Lentivirus of OCN pGreenZeo differentiation reporter-transduced TC28a2 cells were transfected by electroporation or a traditional cationic-lipid transfection reagent with PB-control, PB-LINC02035, or PB-LOC100130207, and the fluorescence was observed under fluorescence microscope 72 h after transfection. The bar graph indicates the fluorescence-based activities of OCN promoter. *P < 0.05 compared with the PB-control group (n = 3 experimental replicates). Scale bars, 200 μm. **(K)** IP/Western blot analysis was performed to investigate whether overexpression of LINC02035 or LOC100130207 affects ubiquitination of RUNX2 protein in TC28a2 cells. RUNX2-FLAG protein was immunoprecipitated using FLAG antibody followed western blot analysis using FLAG and Ubiquitin antibodies to confirm the ubiquitination of RUNX2. *P < 0.05 in comparison with control group (n = 3 experimental replicates). n.s., not significant.

To determine the effects of upregulated lncRNAs, LINC02035 and LOC100130207 on hypertrophic changes in chondrocytes, we overexpressed LINC02035 or LOC100130207 in human primary chondrocytes and TC28a2 cells using the PiggyBac (PB) expression vector system. Before describing the results, we would first like to explain how the vectors were transfected into TC28a2 cells. PB-LOC100130207 was transfected using traditional cationic-lipid transfection reagents in human primary chondrocytes and TC28a2 cells. However, since PB-LINC02035 was not transfected in case of using traditional methods such as calcium or cationic-lipid transfection reagents (data not shown), the vector was transfected into human primary chondrocytes and TC28a2 cells using an electroporation method. For this reason, in the overexpression experiments, experiments on both lncRNAs were performed independently. Overexpression of LINC02035 did not affect the mRNA expression of *RUNX2* in human primary chondrocytes (Figure 5A), whereas RUNX2 protein level was upregulated in human primary chondrocytes transfected with PB-LICN02035 even though the cells were cultured in non-hypertrophic and non-inflammatory environments (Figure 5B). In addition, the protein levels of hypertrophic markers, such as IHH, COL10A1, and MMP13, were also increased, while the protein levels of COL2A1 and ACAN were slightly decreased (Figure 5B). Similar changes in these mRNA and protein expression levels were observed in TC28a2 cells transfected with PB-LINC02035 (Figure 5C-D). Similarly, overexpression of LOC100130207 did not affect the mRNA expression of *RUNX2*, but produced significant changes in the protein expression level, upregulating hypertrophic markers, but downregulating COL2A1 and ACAN proteins in both human primary chondrocytes and TC28a2 cells (Figure 5E-H). In addition, the promoter activity of OCN was significantly increased by overexpressing LINC02035 or LOC100130207 in TC28a2 cells (Figure 5I-J). Moreover, the ubiquitination of RUNX2 protein was clearly decreased in TC28a2 cells transfected with PB-LINC02035 or PB-LOC100130207 (Figure 5K), indicating that both lncRNAs are involved with deubiquitination-mediated stabilization of RUNX2 protein.

### LINC02035 or LOC100130207-mediated phenotypic changes in chondrocytes depend on the presence or absence of RUNX2

**Figure 6:**
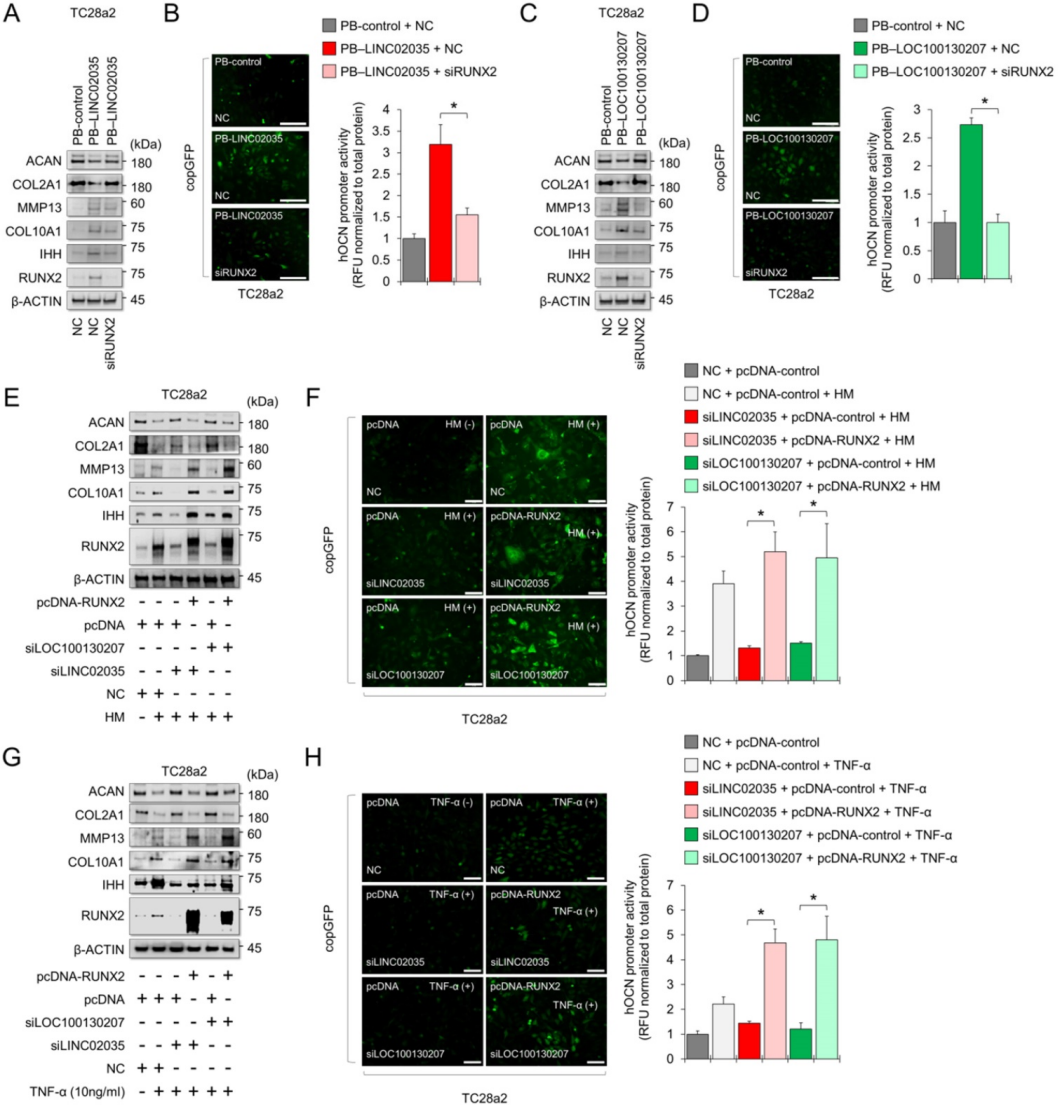
** LINC02035- or LOC100130207-mediated phenotypic changes in chondrocytes are abolished by modulating *RUNX2* expression. (A)** Representative images of western blot analysis for protein levels of RUNX2, IHH, COL10A1, MMP13, COL2A1, and ACAN in TC28a2 cells co-transfected with PB-control or PB-LINC02035 (electroporation) / negative control (NC) siRNA or siRUNX2 (a traditional cationic-lipid transfection reagent). Western blot analysis was performed 72 h after transfection. **(B)** Fluorescence images showing the activity of OCN promoter and the OCN promoter reporter quantity. Bar graph indicates the fluorescence-based activities of OCN promoter. *P < 0.05 compared with the control group (n = 3 experimental replicates). Scale bars, 200 µm.** (C)** Representative images of western blot analysis for protein levels of RUNX2, IHH, COL10A1, MMP13, COL2A1, and ACAN in TC28a2 cells co-transfected with PB-control or PB-LOC100130207/negative control (NC) siRNA or siRUNX2 using a traditional cationic-lipid transfection reagent. Western blot analysis was performed 72 h after transfection. **(D)** Fluorescence images showing the activity of OCN promoter and the OCN promoter reporter quantity. Bar graph indicates the fluorescence-based activities of OCN promoter. *P < 0.05 compared with the control group (n = 3 experimental replicates). Scale bars, 200 µm.** (E)** Representative images of western blot analysis for protein levels of RUNX2, IHH, COL10A1, MMP13, COL2A1, and ACAN in TC28a2 cells co-transfected with pcDNA3.1-mock or pcDNA3.1-RUNX2-FLAG / NC, siLINC02035, or siLOC100130207 using a traditional cationic-lipid transfection reagent. The transfected cells were then treated with hypertrophic medium (HM) for 3 days. Western blot analysis was performed 72 h after treatment of HM. **(F)** Fluorescence images showing the activity of OCN promoter and the OCN promoter reporter quantity. Bar graph indicates the fluorescence-based activities of OCN promoter. *P < 0.05 compared with the control group (n = 3 experimental replicates). Scale bars, 100 µm.** (G)** Representative images of western blot analysis for protein levels of RUNX2, IHH, COL10A1, MMP13, COL2A1, and ACAN in TC28a2 cells co-transfected with pcDNA3.1-mock or pcDNA3.1-RUNX2-FLAG/NC, siLINC02035, or siLOC100130207 using a traditional cationic-lipid transfection reagent. The transfected cells were then treated with TNF-α (10 ng/mL) for 3 days. Western blot analysis was performed 72 h after treatment of TNF-α. **(H)** Fluorescence images showing the activity of OCN promoter and the OCN promoter reporter quantity. Bar graph indicates the fluorescence-based activities of OCN promoter. *P < 0.05 compared with the control group (n = 3 experimental replicates). Scale bars, 100 µm.

To conclude whether both lncRNA-mediated phenotypic changes were due to the stabilization of RUNX2 protein, we performed a rescue study using siRNA or overexpression vector targeting *RUNX2*. The results showed that LINC02035 overexpression-mediated upregulation of hypertrophy markers, including RUNX2, was abolished by the co-transfection of siRNA targeting *RUNX2* in TC28a2 cells, whereas LINC02035-mediated reduction of COL2A1 and ACAN protein levels was rescued by RUNX2 KD (Figure 6A). Likewise, the LINC02035-mediated increase of OCN promoter activity was reduced by *RUNX2* KD (Figure 6B). Similar results were observed in PB-LOC100130207-transfected TC28a2 cells with *RUNX2* KD (Figure 6C-D). Next, we evaluated the effect of RUNX2 overexpression in TC28a2 cells transfected with siRNA targeting LINC02035 or LOC100130207 cultured in hypertrophic culture medium. We found that the hypertrophy marker proteins reduced by siRNA targeting LINC02035 or LOC100130207 were rescued by RUNX2 overexpression, whereas the protein levels of COL2A1 and ACAN recovered by siRNA targeting each lncRNA were increased by RUNX2 overexpression (Figure 6E). Likewise, the promoter activity of OCN was rescued by RUNX2 overexpression (Figure 6G). Similar results were observed in the siRNA-transfected TC28a2 cells cultured with TNF-α (Figure 6H-I). Therefore, we concluded that LINC02035- and LOC100130207-mediated ubiquitination of RUNX2 contributes to chondrocyte hypertrophy in osteoarthritic environments. To determine whether there is the possibility of a positive feedback loop between RUNX2 and both lncRNAs during chondrocyte hypertrophy, qPCR analysis was performed using primary human chondrocytes with RUNX2-KD or RUNX2 overexpression. The results showed that either RUNX2 KD or overexpression did not affect the expression of LINC02035 or LOC100130207 (Figure S2), suggesting that both lncRNAs could only act as upstream regulators for stabilization of RUNX2 protein in chondrocyte hypertrophy.

### Downregulation of LINC02035 and LOC100130207 prevents hypertrophic changes during MSC differentiation to the chondrogenic lineage

MSCs have great potential for regenerating damaged cartilage tissues 25; however, there are obstacles to their clinical application. In particular, chondrocyte hypertrophy is an essential process for endochondral ossification, which can be a problem with the regeneration of articular cartilage, where chondrocytes maintain stable characteristic 26. Therefore, successful cartilage regeneration can be achieved by using chondrogenic MSCs without a hypertrophic phenotype. First, we checked the mRNA and protein expression levels of mRNA and protein for chondrogenic and hypertrophic markers. We confirmed that the mRNA and protein levels of chondrogenic (SOX9 and COL2A1) and hypertrophic markers (COL10A1) clearly increased during the differentiation period, but in RUNX2, the protein level was continuously upregulated during the differentiation period, while the mRNA level showed a tendency to decrease back to the basal level in the late stage of differentiation (Figure 5A-B). Interestingly, the expression levels of both LINC02035 and LOC100130207 were upregulated only in the late stage of differentiation regardless of the pattern of mRNA or protein level of RUNX2 (Figure 5C). To evaluate whether both lncRNAs could influence hypertrophic phenotypes in chondrogenic differentiation of MSCs, we knocked down LINC02035 and LOC100130207 using siRNA and examined their associated phenotypes. We confirmed that both lncRNAs were well reduced by each siRNA, and it was confirmed that the mRNA level of COL10A1 was also reduced, however the mRNA level of RUNX2 was not changed (Figure 5D). As a result of staining with safranin O and alcian blue, using the chondrogenic mass that had been differentiated, the KD of the two lncRNAs had no significant effect on the synthesis of glycosaminoglycans, however the protein level of RUNX2 was barely detected in the siRNA-transfected groups (Figure 5E). Interestingly, the size of the chondrogenic mass transfected with each siRNA was larger than that of the control group. Western blot results showed that KD of LINC02035 or LOC100130207 prevented the upregulation of hypertrophic markers RUNX2 and COL10A1, whereas protein levels of chondrogenic markers SOX9 and COL2A1 were almost unchanged in the siRNA-transfected groups (Figure 5F). These results suggest that LINC02035 and LOC100130207 are novel therapeutic targets for generating MSC-derived chondrogenic masses without hypertrophic phenotypes.

## Discussion

In addition to the degeneration and inflammatory response of articular cartilage, hypertrophic changes in chondrocytes present in articular cartilage have also been identified as hallmark of OA 27. Hypertrophic chondrocytes residing in articular cartilage produce cartilage-degrading enzymes, such as MMP13 and ADAMTS5, which contribute to cartilage destruction and OA development 27-29. Thus, preventing or delaying chondrocyte hypertrophy-like changes might be helpful for further OA progression. However, most studies on OA treatment have mainly focused on the direct control of the inflammatory response 30, 31 or cartilage regeneration using stem cells 32-34. Using anti-inflammatory drugs to suppress the inflammatory response and stem cells to regenerate damaged cartilage tissue can be an excellent OA treatment, but they alone are still insufficient to completely alleviate OA. Therefore, further studies are needed to identify the causes of OA development from diverse perspectives. As mentioned previously, chondrocytes residing in non-calcified areas of articular cartilage should not undergo hypertrophic changes under normal conditions. OA-induced hypertrophic changes in articular chondrocytes can exacerbate OA symptoms and induce vascularization and calcification of the joint cartilage 5. Therefore, suppressing the hypertrophic changes of articular chondrocytes as well as modulating the initial inflammatory responses at the onset of OA would be an important therapeutic strategy for effective treatment to slow down further OA symptoms 35. In this study, we focused on whether inhibiting hypertrophic changes in chondrocytes could prevent the destruction of cartilage tissue due to OA. As shown in Figure 1, the RUNX2 transcription factor may be the most important factor that causes hypertrophic changes in normal chondrocytes. However, little information is available on the expression of RUNX2 in the inflammatory environment caused by OA. Therefore, it is necessary to understand the mechanisms underlying the onset of RUNX2 in osteoarthritic chondrocytes. Interestingly, we found that the expression level of *RUNX2* mRNA remained unchanged during hypertrophic differentiation and inflammatory responses in normal chondrocytes, however the protein level was significantly increased (Figure 1). Furthermore, MG132 treatment increased protein levels of RUNX2 (Figure 4G, 4H). Taken together, these results suggest that RUNX2 is regulated at the post-translational level during hypertrophic changes and inflammatory responses in chondrocytes. Therefore, it would be important for the development of OA therapy, based on the inhibition of chondrocyte hypertrophy, to discover upstream regulators that can be responsible for the post-translational process of RUNX2 and to understand their molecular mechanisms of action.

LncRNAs are characterized according to their structural features and subcellular localization and can be classified as antisense, lincRNA, sense-overlapping, sense intronic, and processed transcripts 36. Because lncRNAs have flexible structural characteristics, they are known to bind to DNA, RNA, and proteins and form an extensive gene expression regulatory network with DNA, RNA, and Protein 37. Accumulating evidence has shown that lncRNAs are involved in cartilage tissue, chondrocyte homeostasis, and chondrogenesis 38. Since OA is characterized by the degradation of articular cartilage, most studies have focused on the function of lncRNAs as important regulators of the maintenance or degradation of the extracellular matrix (ECM) 39-42. RUNX2 also plays an important role in the degradation of articular cartilage through hypertrophy of chondrocytes and by directly regulating the expression of MMP13 43. In this study, we discovered two novel lncRNAs, LINC02035 and LOC100130207, that interact with RUNX2. During hypertrophic changes and inflammatory conditions in chondrocytes, the amounts of LINC02035 and LOC100130207 increased and mainly accumulated around the nucleus of the cells. Interestingly, when the expression of these two lncRNAs was silenced with siRNA, ubiquitination of the RUNX2 protein, which was decreased during chondrocyte hypertrophy or inflammatory response, was not reduced (Figure 4). Therefore, an increase in the non-coding RNAs LINC02035 or LOC100130207 is thought to play a key role in stabilizing RUNX2 protein during chondrocyte hypertrophy. In this study, we did not clarify how the two lncRNAs can bind to the RUNX2 protein and the mechanism of action that can induce stabilization of the RUNX2 protein. However, we not only confirmed through RNA-IP (Figure 2H-J) that the lncRNAs LINC02035 and LOC100130207 were enriched in the RUNX2 protein during the inflammatory response of chondrocytes, but also confirmed that the sequences “TGTGGT” and “AGTGGT,” known as RUNX2 binding motifs 44, 45, were included in these two lncRNAs. Therefore, although further studies are required to clarify the mechanisms of action between the RUNX2 protein and these two lncRNAs, the current study is meaningful in that it discovered novel lncRNAs that are related to chondrocyte hypertrophy and evaluated their function in *in vitro* OA experimental models.

**Figure 7:**
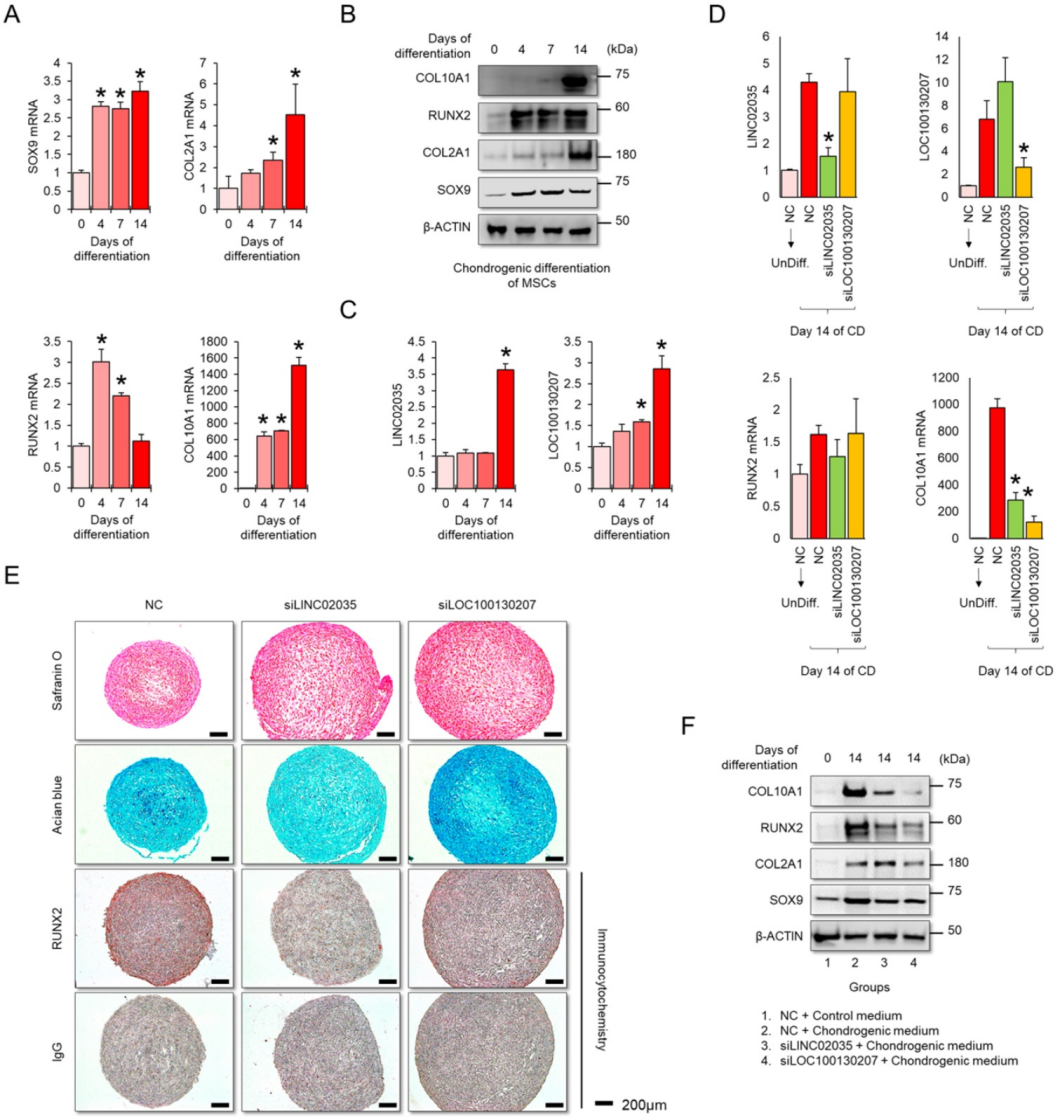
** Knockdown of LINC02035 and LOC100130207 prevents hypertrophic changes during chondrogenic differentiation of MSCs. (A)** qRT-PCR analysis of *SOX9*, *COL2A1*, *RUNX2*, and *COL10A1* mRNAs during chondrogenic differentiation of human bone marrow-derived MSCs. *P < 0.05 in comparison with Day 0 group (n = 3 experimental replicates). **(B)** Representative images of western blot analysis for protein levels of SOX9, COL2A1, RUNX2, and COL10A1 during chondrogenic differentiation of human bone marrow-derived MSCs. β-ACTIN was used as a loading control. **(C)** qRT-PCR analysis of *LINC02035* and *LOC100130207* lncRNAs during chondrogenic differentiation of human bone marrow-derived MSCs. *P < 0.05 in comparison with Day 0 group (n = 3 experimental replicates). **(D)** qRT-PCR analysis of *LINC02035* and *LOC100130207*,* RUNX2*, and *COL10A1* expression during chondrogenic differentiation of human bone marrow-derived MSCs transfected with each targeting siRNA. *P < 0.05 in comparison with NC group (n = 3 experimental replicates). NC, non-targeting control siRNA. **(E)** Proteoglycan staining of chondrogenic mass by safranin O and alcian blue and immunostaining for RUNX2 were performed in chondrogenic micromass cultures derived from human bone marrow-derived MSCs at day 14 of differentiation. Scale bars, 200 µm. **(F)** Representative images of western blot analysis for protein levels of SOX9, COL2A1, RUNX2, and COL10A1 during chondrogenic differentiation of human bone marrow-derived MSCs transfected with siRNAs targeting LINC02035 or LOC100130207. β-ACTIN was used as a loading control.

MSC-based cell therapy has great potential for the regeneration of damaged articular cartilage 25, 46. However, the regeneration of articular cartilage by externally transplanted MSC has limitations, such as chondrocyte hypertrophy which occurs simultaneously during the process of MSC differentiation into chondrogenic lineage. This is undesirable for cartilage regenerative approaches because hypertrophic chondrocytes contribute to the formation of abnormal ECM and calcification of regenerated cartilage tissues 47 as well as the production of cartilage-degrading enzymes, such as MMP13 48. Therefore, using chondrogenic MSCs with a reduced hypertrophic phenotype would be beneficial in MSC-based therapies for cartilage regeneration. Our study showed that siRNA-mediated KD of lncRNAs LINC02035 or LOC100130207 reduced the protein levels of RUNX2 and COL10A1; however, those of SOX9 and COL2A1 were highly detected during *in vitro* chondrogenic differentiation of MSCs (Figure 7), which means that LINC02035- or LOC100130207-deficient chondrogenic MSCs may be more effective for articular cartilage regeneration. It should be noted that we have not been able to conduct *in vivo* experiments using experimental animal models because, unfortunately, LINC02035, which we discovered through this study, has not been found to have interspecies locus conservation in available experimental animal models (*https://lncipedia.org/db/gene/LINC02035*), and LOC100130207 has not yet been characterized (*https://www.ncbi.nlm.nih.gov/gene/100130207#gene-expression*). Protein-coding genes are well conserved among mammals, whereas most human lncRNAs are non-conserved 49-51. The low conservation of these lncRNA genomic sequences between species can bring many limitations to the *in vivo* studies of newly discovered lncRNAs. Despite these limitations, some studies have shown clear effects, at least in *in vitro* cell culture models, although lncRNAs are not sequentially conserved 52, 53. Although information on interspecies lncRNA conservation is not yet fully understood, a recent study has provided a pipeline for *in vivo* experiments on non-conserved lncRNAs in experimental animals 54. If a lot of information on these non-conserved lncRNAs is accumulated over the next several decades, it is thought that pipelines for various *in vivo* experiments will be provided in the future based on these experimental results.

In summary, our findings contribute to the understanding of the mechanisms that regulate an increased level of RUNX2 protein in chondrocytes in *in vitro* OA environments. We demonstrated that the lncRNAs LINC02035 and LOC100130207 were upregulated during hypertrophic differentiation and inflammatory response in chondrocytes, leading to preferential localization and accumulation in the nucleus of these two lncRNAs. Consistent with the results of these experiments in the normal human chondrocyte cell line TC28a2, we demonstrated the co-expression of RUNX2 and these two lncRNAs in primary chondrocytes isolated from the articular cartilage of patients with OA. The RUNX2 protein was deubiquitinated under hypertrophic or inflammatory conditions, and KD of LINC02035 and LOC100130207 led to RUNX2 ubiquitination under the same conditions. Therefore, we suggest that lncRNA LINC02035- or LOC100130207-mediated RUNX2 stabilization may be important regulatory axes of chondrocyte hypertrophy, and these two lncRNAs could be targets for gene therapy that can slow down or prevent the progression of OA by modulating RUNX2-mediated chondrocyte hypertrophy.

## Materials and Methods

### Preparation of human cartilage tissues, primary chondrocytes, TC28a2 cells, and MSCs

Cartilage tissue samples were obtained from three patients with OA who underwent total knee arthroplasty with approval from the Institutional Review Board (IRB) (2019-1374-002) of the Yonsei University College of Medicine. To isolate primary chondrocytes, cartilage tissues were shaved from the articular surface under sterile conditions, minced into as many pieces as possible, and washed three times with phosphate-buffered saline (PBS). The sliced tissues were then incubated in Dulbecco's Modified Eagle's high-glucose medium (DMEM-HG; Invitrogen, Carlsbad, CA, USA) supplemented with 2% collagenase (Sigma-Aldrich, St. Louis, MO, USA) and 0.5% dispase (Sigma-Aldrich) at 37 °C and 5% CO_2_ for 4 h. The cell suspensions were then passed through a 40 μm cell strainer to remove debris, and the cells were cultured in DMEM-HG containing 10% fetal bovine serum (FBS; Gibco, Grand Island, NY, USA) and 1% antibiotic-antimycotic solution (Invitrogen) at 37 °C and 5% CO_2_. The TC28a2 normal human chondrogenic cell line was purchased from Sigma-Aldrich and cultured under the same conditions as the primary chondrocytes. Human bone marrow-derived MSCs were obtained from the posterior iliac crests of three adult donors with the approval of the IRB (2017-0308-001) of the Yonsei University College of Medicine. MSCs isolated from the bone marrow were selected based on their ability to adhere to plastic cell culture plates, and we confirmed that all cultured cells (98%) were positive for CD90 and CD105 and negative for CD34 and CD45, as described previously^55^. MSCs were cultured in DMEM-low glucose (LG; Invitrogen) containing 10% FBS and 1% antibiotic-antimycotic solution at 37 °C and 5% CO_2_.

### Reagents and differentiation methods

IL-1β (R&D Systems Inc., Minneapolis, MN, USA), TNF-α (R&D Systems Inc.), and IL-6 (Koma Biotech, Seoul, South Korea) were used to create an inflammatory environment in chondrocytes. IL-1β and TNF-α were used at a concentration of 10 ng/ml based on our previously published study 56 and IL-6 was used at a concentration of 50 ng/ml based on our previous study 57. MG-132 (proteasome inhibitor; Sigma-Aldrich) was used to confirm whether RUNX2 is regulated at the post-translational level in normal chondrocytes. To induce hypertrophic differentiation of chondrocytes, we referred to the differentiation medium composition described in a paper published by another group 18. Briefly, TC28a2 cells were cultured in DMEM-HG supplemented with 1% FBS, 1% insulin transferrin selenium-A (ITS, Invitrogen), 100 ng/ml BMP-2 (R&D Systems Inc.), 10 ng/ml GDF-5 (PeproTech, Rosemont, IL, USA), 1 μM 3,3,5-Triiodo-L-thyronine (T3) (Sigma-Aldrich), 50 μg/ml L (+)-ascorbic acid (Sigma-Aldrich), 10 nM dexamethasone (Sigma-Aldrich), 10 mM β-glycerophosphate (Sigma-Aldrich), and 1% antibiotic-antimycotic solution. The hypertrophic medium was replaced every 2 days for 5 days. To induce *in vitro* chondrogenic differentiation of MSCs, a micromass culture method was employed. MSCs were trypsinized and resuspended in DMEM-HG at a density of 1 × 10^7^ cells/ml. The cells were dotted on the center of individual wells of 24-well plates at 1 × 10^5^ cells per well in a 10 μl volume. The cells were allowed to adhere for 2 h in the cell culture incubator, and then chondrogenic medium, consisting of DMEM-HG supplemented with 1% antibiotic-antimycotic solution, 1% insulin transferrin selenium-A (ITS; Invitrogen), 50 mg/ml ascorbic acid (Sigma-Aldrich), 40 μg/ml L-Proline (Sigma-Aldrich), and 10 ng/ml TGF-b3 (R&D Systems Inc.). The chondrogenic medium was changed every two days for 14 days. Safranin O and alcian blue staining were performed to evaluate the chondrogenic potential and proteoglycan synthesis of the MSCs. Chondrogenic mass pellets were washed with PBS and fixed in 10% formalin for approximately 24 h. After fixation, the pellets were dehydrated in ethanol, and the dehydrated pellets were paraffin-embedded and sectioned. The micromass sections were deparaffinized and stained with safranin O (1% in acetic acid solution; Sigma-Aldrich) or alcian blue (0.3% in 70% ethanol; Sigma-Aldrich) for 30 min. The stained pellet sections were mounted and evaluated microscopically.

### Immunostaining

Joint cartilage tissues were fixed with 10% formalin at room temperature and formalin-fixed specimens were embedded in paraffin. The paraffin-embedded sections were deparaffinized, rehydrated, washed with PBS, and the tissue sections were used to evaluate the disruption of cartilage tissue and to detect chondrocyte hypertrophy-related marker proteins in human cartilage tissues. The prepared tissue samples were sliced to a thickness of 4 μm and probed with anti-IHH (Santa Cruz Biotechnology, Santa Cruz, CA, USA), anti-RUNX2 (Santa Cruz Biotechnology), or anti-COL10A1 (Santa Cruz Biotechnology) antibody to detect IHH, RUNX2, or COL10A1 levels, respectively. To visualize each target protein in the anti-IHH-, anti-RUNX2-, or anti-COL10A1 antibody-probed tissues, the samples were stained with aminoethylcarbazole (AEC; Abcam, Cambridge, UK). The stained tissues were observed using a VS120 virtual microscope (Olympus, Tokyo, Japan) and images of the sections were analyzed using the OlyVIA 2.5 program (Olympus). For immunocytochemistry, human articular chondrocytes were seeded at approximately 5000 cells/cm^2^ on 4-well chamber slides (Nalge Nunc International, Rochester, NY, USA) and incubated in a 5% CO_2_ incubator at 37 °C for an overnight incubation. The cells were then washed with PBS followed by fixation with 4% paraformaldehyde (Sigma) for 30 min. 1% Triton X-100 in PBS was employed for cell permeabilization and then the cells were incubated with 3% bovine serum albumin (BSA) in PBS for 60 min. After then, the cells were incubated with a 1 : 200 dilution of primary antibodies against RUNX2 (Santa Cruz Biotechnology) overnight at 4 °C. After washing with PBS, the cells were incubated with phycoerythrin conjugated secondary antibody (Santa Cruz Biotechnology) in 1 : 5000 dilution in 3% BSA-containing PBS for 60 min at room temperature in the dark. After washing two times with PBS, the cells were incubated with Alexa Fluor 488 Phalloidin (Invitrogen) to visualize cellular cytoskeleton. The nuclei were stained with 4,6-diamidino-2-phenyindole (DAPI, Sigma) and then examined using a Zeiss LSM700 scanning laser confocal microscope (Zen 2011; Carl Zeiss MicroImaging GHBH, Jena, Germany).

### qPCR

qPCR was performed as described previously 58. Briefly, total RNA was isolated using an RNeasy kit (Qiagen, Valencia, CA, USA), according to the manufacturer's instructions. Total RNA (1 μg of total RNA) was reverse-transcribed using an Omniscript kit (Qiagen, Hilden, Germany). Primer sets used in this study were validated and purchased from Bioneer (Daejeon, South Korea). The validated and designed primer sets are listed in Table S5. The mean cycle threshold values from triplicate measurements were used to calculate gene expression, with normalization to ACTB (β-actin) as the internal control.

### Western blot analysis

TC28a2 cells or MSCs were lysed in PROPREP^TM^ protein extraction solution (iNtRON Biotechnology, Seongnam, South Korea). Protein concentrations were determined using the Bio-Rad Protein Assay (Bio-Rad Laboratories, Inc., Hercules, CA, USA). Approximately 20-30 μg of protein was analyzed by 10% SDS-polyacrylamide gel electrophoresis (SDS-PAGE; Sigma-Aldrich). The resolved proteins were transferred to membranes and blocked with 5% skim milk (BD, Sparks, MD, USA) for 1 h at room temperature. Membranes were incubated overnight with antibodies against COX2 (1:1000; BD Biosciences, San Jose, CA, USA), IHH (1:1000; Santa Cruz Biotechnology), RUNX2 (1:1000; Santa Cruz Biotechnology), COL10A1 (1:1000; Abcam, Cambridge, UK), COL2A1 (1:1000; Santa Cruz Biotechnology), ACAN (1:1000; Santa Cruz Biotechnology), MMP13 (1:1000; Cell Signaling Technology, Danvers, MA, USA), ubiquitin (1:1000; Santa Cruz Biotechnology), SOX9 (1:1000; Santa Cruz Biotechnology), FLAG (1:1000; Sigma-Aldrich), and HSP90 (1:5000; Santa Cruz Biotechnology). The membranes were further probed with antibodies against β-actin (1:5000; Santa Cruz Biotechnology), which served as a loading control.

### Nuclear and cytoplasmic fractionation

Human primary chondrocytes isolated from intact or damaged cartilage obtained from OA patients were collected by scrapping and washed twice with PBS prior to nuclear and cytosolic protein fractionation. Nuclear and cytoplasmic fractionation were conducted using the NE-PER Nuclear and Cytoplasmic Extraction Reagents Kit (Thermo Fisher Scientific, Rockford, IL, USA) according to the manufacturer's instructions. Each separated protein was used for detection of RUNX2 protein by western blot analysis.

### Human OCN pGreenZeo differentiation reporter assay

To establish TC28a2 cells that stably express copGFP where the OCN promoter/enhancer is active, the lentiviral plasmid vector for the human OCN pGreenZeo differentiation reporter was purchased (System Biosciences, LLC; SBI SR1003PA-1). HEK293T cells were seeded in T75 flasks at a density of 5 × 10^6^ cells per dish to obtain lentiviral particles with a human OCN pGreenZeo differentiation reporter. The attached cells were transfected with a lentiviral plasmid for the human OCN pGreenZeo differentiation reporter with psPAX2 (*http://www.addgene.org/12260/*) and pMD2.G (http://www.addgene.org/12259/) using CalFectin mammalian cell transfection reagent (SignaGen Laboratories, Frederick, MD, USA). The medium was gently replaced approximately 6 h after transfection. Transfected cells were maintained for 2 days, and the supernatants were collected and stored at -70 °C. To monitor GFP activity in TC28a2 cells transduced with lentivirus of the OCN pGreenZeo differentiation reporter, the cells were seeded in 6-well plates at a density of 1 × 10^5^ cells under hypertrophic differentiation or inflammatory conditions. After 5 days of hypertrophic differentiation or inflammation induction, copGFP signals were observed using fluorescence microscopy. For quantitative analysis, the passive lysis buffer (Promega, Madison, WI, USA) was treated with the cells, which were incubated at 4 °C for 5 min. The whole lysate was transferred to a microcentrifuge tube and centrifuged to collect the supernatant. After determining the total protein concentration of each lysate sample, fluorescence was measured using a fluorescence plate reader at 360 nm/465 nm (excitation/emission).

### RNA interference (RNAi)

All siRNAs used in this study were purchased from Bioneer. Information regarding the siRNAs used in this study is provided in Table S6. Briefly, TC28a2 cells or MSCs were plated to obtain 70-80% confluent growth in 6-well plates and transfected with 100 nM of each siRNA using Lipofectamine 2000 (Invitrogen). After 6 h of transfection, the medium was replaced with a fresh medium. The next day, the cells were detached from the plates, counted, and reseeded at appropriate cell densities for each experiment.

### RNA sequencing and data analysis

To prepare samples for RNA sequencing, total RNAs was extracted from TC28a2 cells treated with growth medium (n = 2) or hypertrophic medium (n = 3) on day five of differentiation. RNA was extracted using TRIzol (Invitrogen) following the protocols provided by the manufacturer. Isolated RNAs were submitted to Macrogen Inc. (Seoul, South Korea) for total RNA sequencing. The overall quality of the extracted total RNAs was validated using spectrophotometry. To remove low-quality and adapter sequences, the raw reads were read by the sequencer before analysis, and the processed reads were aligned to Homo sapiens using HISAT2 v2.1.0 59. The reference genome sequence of Homo sapiens (hg19) and annotation data were downloaded from NCBI, and transcript assembly of known transcripts was then processed using StringTie v2.1 60, 61. Based on these results, transcript and gene expression levels were calculated as read count or fragments per kilobase of exon per million fragments mapped (FPKM) value per sample. The expression profiles were used to perform additional analyses, such as determining the number of differentially expressed genes (DEGs). The relative abundance of genes was measured in read counts using StringTie. Genes with one or more than zero read count values in the samples were excluded. The filtered data were log2-transformed and subjected to RLE normalization. The statistical significance of the differential expression data was determined using the DESeq2 nbinomWaldTest 62 and fold change, in which the null hypothesis was that no difference exists among groups. The false discovery rate (FDR) was controlled by adjusting the p-value using the Benjamini-Hochberg algorithm. For the DEG set, hierarchical clustering analysis was performed using complete linkage and Euclidean distance as similarity measures. Gene ontology analysis was performed for DEGs using the g:Profiler 63 and KEGG pathway analysis was performed based on the KEGG pathway (https://www.genome.jp/kegg/) database. We used the multidimensional scaling (MDS) method to visualize similarities among samples. We applied Euclidean distance as a measure of dissimilarity. Hierarchical clustering analysis was also performed using complete linkage and Euclidean distance as a measure of similarity to display the expression patterns of differentially expressed transcripts that were satisfied with a fold change ≥ 2 and raw P < 0.05. All data analyses and visualization of differentially expressed genes were conducted using R (version 3.6.1; *www.r-project.org*). RNA sequencing data are available in the GEO database with the accession number GSE199847, and differential expression information for each mRNA and lncRNA is listed in Table S1-4.

### RNA-IP

RNA-IP was described previously 64. Briefly, RNA-IP was performed to determine the potential interactions between RUNX2 protein and the lncRNAs selected in this study. TC28a2 cells (1 × 10^7^) transfected with the pcDNA3.1-RUNX2 vector were used for each experiment. RUNX2-bound RNA-IP was performed using the RiboCluster Profiler RIP assay kit protocol (MBL International Corporation, Woburn, MA, USA). The cells were lysed using dithiothreitol (DTT; Sigma)-added lysis buffer provided by the manufacturer, and then the lysates were precleared with protein G plus agarose beads (Thermo Fisher Scientific) in DTT-added wash buffer provided by the manufacturer. The precleared lysates were transferred to prepared tubes containing RUNX2 antibody (Santa Cruz Biotechnology) or IgG-immobilized beads. After overnight incubation, the RUNX2 antibody or IgG-immobilized protein G agarose beads-RNA/protein complexes were separated to extract proteins and PUM1-bound RNAs. The eluted RNA was analyzed by qPCR. RUNX2 antibody-bound RNAs were isolated and purified using a kit according to the manufacturer's instructions.

### Enzyme-linked immunosorbent assay (ELISA) for RUNX2 activity

The activity measurement of RUNX2 was performed using an ELISA kit for human runt-related transcription factor 2 (Cloud-Clone Corp., Katy, TX, USA) according to the manufacturer's instructions. Briefly, TC28a2 cells were lysed before the assay according to the manufacturer's instructions. Samples used for this study or standards provided by the manufacturer were transferred to antibody-pre-coated plates and the samples were incubated for 1 h at 37 °C. After aspirating each sample, the prepared detection reagent A was added, and the plates were incubated for 1 h at 37 °C. After washing, detection reagent B was added and the plates were incubated for 30 min at 37 °C. The solution in the plate was aspirated and washed with wash buffer provided by the manufacturer. Afterwards, substrate solution was added to the plates, and the plates were incubated for 20 min at 37 °C. After addition of the stop solution, the plate was immediately read using a spectrophotometric microplate reader at 450 nm. All samples and standards were tested in triplicate.

### lncRNAs LINC02035 and LOC100130207 *in situ* hybridization

The cellular localization and *in situ* visualization of LINC02035 and LOC1001303207 were observed using an RNAscope 2.5 HD Assay-RED kit (Advanced Cell Diagnostics, Hayward, CA, USA) according to the manufacturer's instructions. For sample preparation, articular cartilage tissues obtained from OA patients undergoing total knee arthroplasty were shaved from the articular surface under sterile conditions, divided into intact or damaged sections, and completely minced. We then isolated Primary chondrocytes from intact or damaged cartilage, respectively, and allowed the cells to attach to 4-well glass chamber slides (Nalge Nunc International, Rochester, NY, USA) in 5% CO_2_ incubator at 37 °C overnight. The next day, the cells were immediately used for the RNA *in situ* hybridization assay. Standard positive control (Hs-PPIB, ACD-313901) and negative control (DapB, ACD-310043) probes were used to ensure interpretable results. Probes for *in situ* hybridization of LINC02035 (Hs-LINC02035, ACD-1116341-C1) and LOC100130207 (Hs-LOC100130207-C1, ACD-1116351-C1) were also manufactured and purchased from Advanced Cell Diagnostics. The sequence information for each probe is as follows: Hs-LINC02035-C1 [Cat. No. 1116341; accession no. NR_024618.1; No. of pairs 20; target region 1451-2476] and Hs-LOC100130207-C1 [Cat. No. 1116351; accession no. NR_149025.1; No. of pairs 20; Target region 645-1765]. All experimental procedures for RNA *in situ* hybridization were performed in strict accordance with the manufacturer's instructions. Briefly, cells seeded on 4-well glass chamber slides were fixed in 10% neutral buffered formalin, and the slides were treated with peroxidase block solution for 15 min at room temperature. Afterwards, the retrieval solution was treated to each slide for 15 min at 100 °C. The hybridization steps were conducted in a drying oven provided by Advanced Cell Diagnostics for 2 h at 40 °C. After the washing step, samples were incubated with signal amplification solution as follows: Amp1 for 30 min at 40 °C, Amp2 for 15 min at 40 °C, Amp3 for 30 min at 40 °C, Amp4 for 15 min at 40 °C, Amp5 for 30 min at room temperature, and Amp6 for 15 min at room temperature. The samples were then incubated with Fast Red A and B solutions to visualize each target lncRNA-positive dot and with hematoxylin for counterstaining. The results were observed using a standard bright-field microscope.

### RNA subcellular fractionation

Fractionation of cytoplasmic and nuclear RNAs in TC28a2 cells was performed using an RNA Subcellular Isolation Kit (Active Motif, Inc., Carlsbad, CA, USA) according to the manufacturer's instructions. Isolated cytoplasmic and nuclear RNA from TC28a2 cells were reverse transcribed, and the expression levels of each lncRNA selected in this study were evaluated by qPCR. To obtain the percentages of cytoplasmic and nuclear RNA, normalization of the data was performed against total cellular RNA [% of input = 100 × [2^ (Ct total RNA - Ct RNA fraction)]].

### IP

Lysates from TC28a2 cells transfected with pcDNA3.1-RUXN2 or siRNAs targeting LINC02035 or LOC100130207 were prepared with non-denaturing lysis buffer, as previously described 58. The lysates were incubated with protein A/G agarose beads (Santa Cruz Biotechnology) and antibodies against RUNX2 (Santa Cruz Biotechnology). Beads conjugated with both lysates and RUNX2 antibody were collected after centrifugation and washed three times with lysis buffer. The complexes were released from the beads by boiling with 2× SDS sample dye and western blotting was performed. All membranes were incubated with antibodies against RUNX2 (Santa Cruz Biotechnology), ubiquitin (Santa Cruz Biotechnology), or HSP90 (Santa Cruz Biotechnology) for 12 h, followed by incubation with an HRP-conjugated secondary antibody for 1 h.

### Overexpression of LINC02035 or LOC100130207

PiggyBac (PB) control, PB-LINC02035, and PB-LOC100130207 expression vectors were designed, synthesized, and purchased from VectorBuilder (VectorBuilder Inc, Chicago, IL, USA). Information of the vector used in this study is shown in Supplementary Figure S1. PB-control or PB-LINC02035 was transfected with TC28a2 cells using electroporation with the neon transfection system (Cat no, MPK5000; Invitrogen), and the electroporation method was described previously 64. Briefly, TC28a2 cells were prepared at approximately 1 × 10^6^ cells per trial, suspended in 90 µL of Transfection Resuspension Buffer, and then mixed with 6 μg of plasmid DNA in 10 µL volume. Next, the mixture was placed in the electroporator Neon Gold Pipette Tips and then pulsed (Pulse voltage: 990 V, pulse width: 40 ms). For transfection of PB-control or PB-LOC100130207 vector, TC28a2 cells were plated to obtain 70-80% confluent growth in 6-well plates and transfected with 1 μg of each plasmid vector per well using Lipofectamine 2000 (Invitrogen). Transfection efficiency was confirmed by qPCR 48 h after electroporation or transfection.

### Statistics and reproducibility

All experiments were performed in triplicates using samples from at least three donors except for the control group (n = 2) in RNA-sequencing. A Student's t-test was used to assess the differences between the two groups. The statistical significance of the differences between three or more groups was calculated using a one-way analysis of variance (ANOVA) and post-hoc Bonferroni correction. Data are presented as mean ± standard deviation. For all tests, P < 0.05 was considered statistically significant. Interaction strength was predicted using RPISeq (*http://pridb.gdcb.iastate.edu/RPISeq/index.html*).

Exclusion criteria for cartilage tissues obtained from OA patients undergoing total knee arthroplasty were a history of rheumatoid arthritis, inflammatory arthritis, autoimmune disease, ankylosing spondylitis, cancer, and long-term corticosteroid therapy.

All experiments performed in this study included at least two or three biological replicates; the number of replicates is mentioned in the text or figure legends.

## Supplementary Material

Supplementary figures and tables 5-6.

Supplementary table 1.

Supplementary table 2.

Supplementary table 3.

Supplementary table 4.

## Abbreviations

ACAN: aggrecan
ANOVA: analysis of variance
BMP-2: bone morphogenic protein-2
cKO: conditional knockout
COL2A1: collagen, type II, alpha 1
COL10A1: collagen, type X, alpha 1
copGFP: copepod green fluorescent protein
DapB: bacillus subtilis dihydrodipicolinate reductase
DMEM-HG: dulbecco's modified eagle's high-glucose medium
DMEM-LG: dulbecco's modified eagle's low-glucose medium
DMM: destabilization of the medial meniscus
ELISA: enzyme-linked immunosorbent assay
FBS: fetal bovine serum
FDR: false discovery rate
FOXO: forkhead box transcription factors
GDF-5: Growth/differentiation factor 5
GFP: green fluorescent protein
HSP90: heat shock protein 90
Hs-PPIB: human peptidyl-prolyl cis-trans isomerase B
IHC: immunohistochemistry
IHH: Indian hedgehog
IL-1β: interleukin-1β
IL-6: interleukin-6
IP: immunoprecipitation
KD: knockdown
KEGG: Kyoto encyclopedia of genes and genomes
LncRNA: long-non-coding RNA
MG132: carbobenzoxy-leu-leu-leucinal
MMP13: matrix metallopeptidase 13
MSC: mesenchymal stem cell
OA: osteoarthritis
OCN: osteocalcin
PB: PiggyBac
PBS: phosphate-buffered saline
PI3K-AKT: phosphatidylinositol-3-kinase and protein kinase B
PTGES: prostaglandin E synthase
qPCR: quantitative polymerase chain reaction
RNA-IP: RNA-immunoprecipitation
RUNX2: runt-related transcripion factor 2
SDS: sodium dodecyl sulfate
SOX9: sex-determining region Y-box 9
TNF-α: tumor necrosis factor-α
T3: 3,3,5-triiodo-L-thyronine
