# Supplementary material for: RUNX2 stabilization by long non-coding RNAs contributes to hypertrophic changes in human chondrocytes
Source: Int J Biol Sci. 2023 Jan 1;19(1):13–33. doi: 10.7150/ijbs.74895 (PMC9760429; doi:10.7150/ijbs.74895)
Supplement: Supplementary file 1 — Supplementary figures and tables 5-6. [file ijbsv19p0013s1.pdf]

SUPPLEMENTARY MATERIAL

RUNX2 stabilization by long non-coding RNAs contribute to hypertrophic changes in human chondrocytes

Dong Suk Yoon[1], Eun-Ji Kim[1,2], Sehee Cho[1,2], Soyeong Jung[1,2], Kyoung-Mi Lee[1,3], Kwang Hwan Park[1], Jin Woo Lee[1,2,3*], Sung-Hwan Kim[1,4,5*]

[1]Department of Orthopaedic Surgery, Yonsei University College of Medicine, Seoul 03722, South Korea

[2]Brain Korea 21 PLUS Project for Medical Science, Yonsei University College of Medicine, Seoul 03722, South Korea

[3]Severance Biomedical Science Institute, Yonsei University College of Medicine, Seoul 03722, South Korea.

[4]Arthroscopy and Joint Research Institute, Yonsei University College of Medicine, Seoul 03722, South Korea

[5]Department of Orthopedic Surgery, Gangnam Severance Hospital, Yonsei University College of Medicine, Seoul 06273, South Korea

[*]Correspondence: Jin Woo Lee (ljwos@yuhs.ac) or Sung-Hwan Kim (orthohwan@gmail.com)

Running title: RUNX2-interacting lncRNAs are involved in chondrocyte hypertrophy

-Supplementary figures-

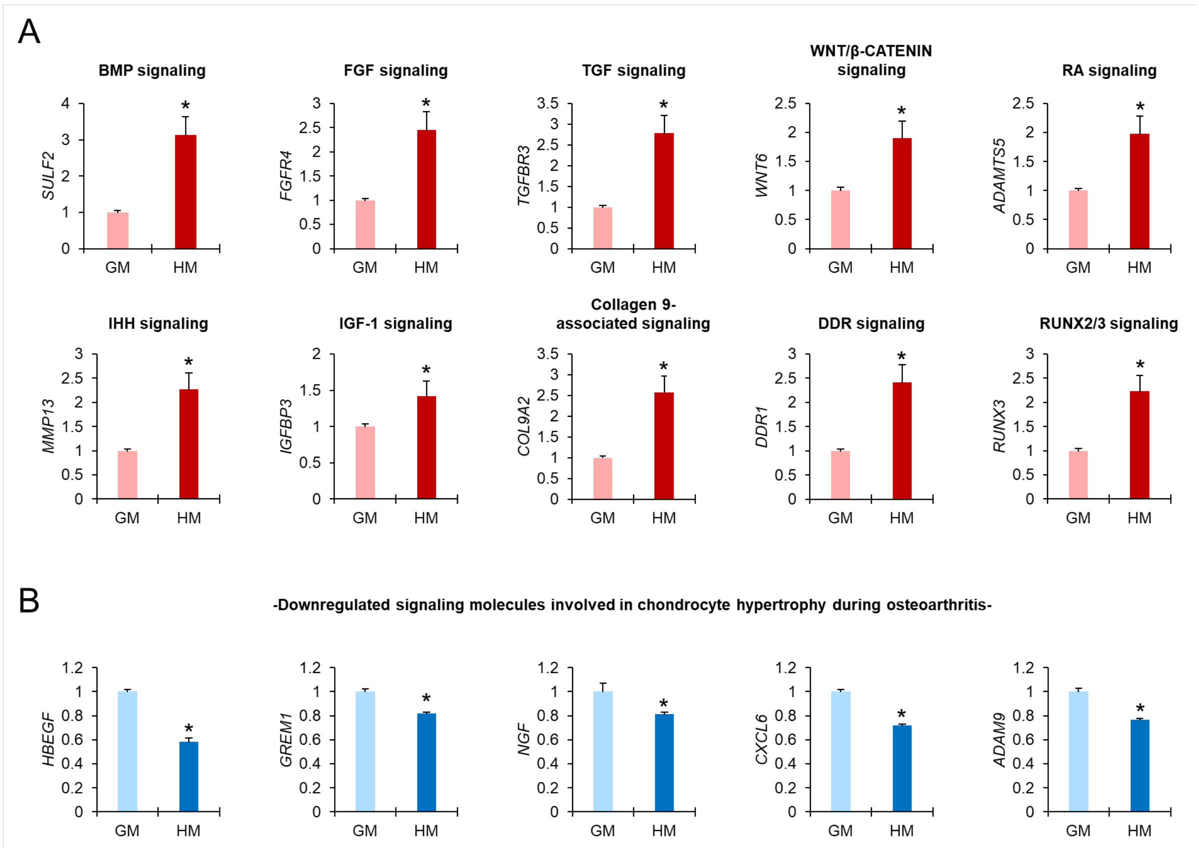

Supplementary Fig. S1. qPCR confirmation for RNA-sequencing. (**A**) Validation of 10 selected upregulated mRNAs and (**B**) 5 selected downregulated mRNAs. For more details, please see Table S1 and S2.

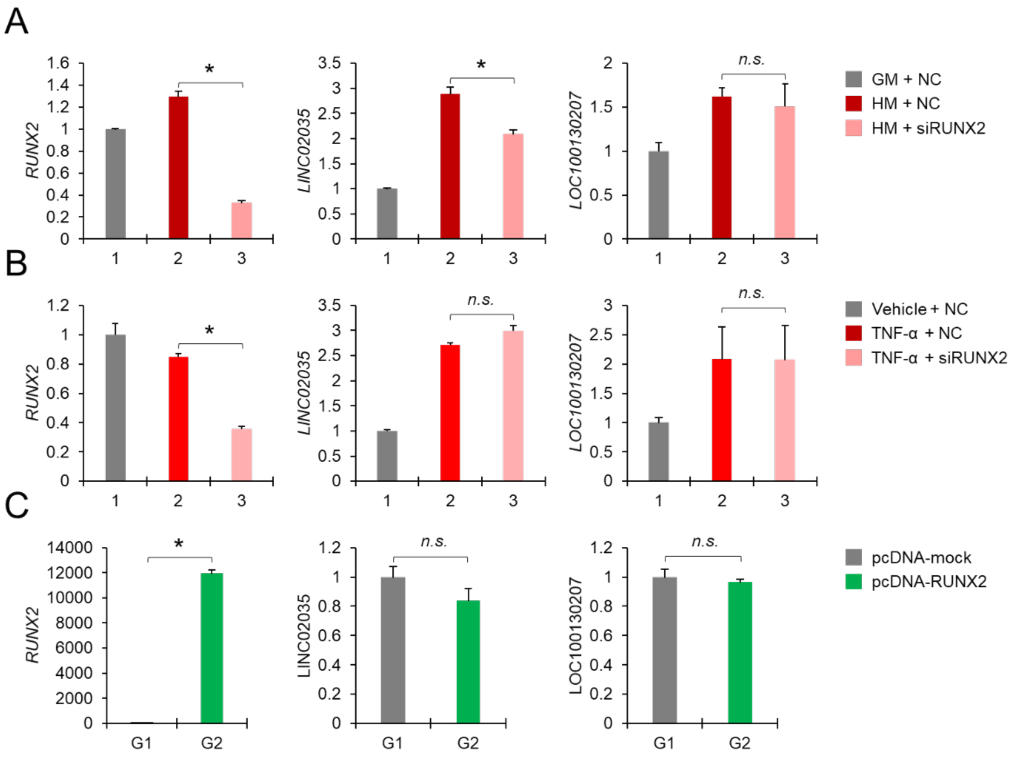

Supplementary Fig. S2. Effects of RUNX2 downregulation or overexpression on expression of LINC02035 or LOC100130207 in human primary chondrocytes. qPCR analysis was performed to determine the effects of RUNX2 knockdown on upregulated expression of LINC02035 or LOC100130207 in (**A**) hypertrophy-induced medium (HM)- or (**B**) TNF-α-treated human primary chondrocytes. (**C**) Conversely, qPCR analysis was performed to determine the effects of RUNX2 overexpression on the expression of both lncRNAs in human primary chondrocytes.

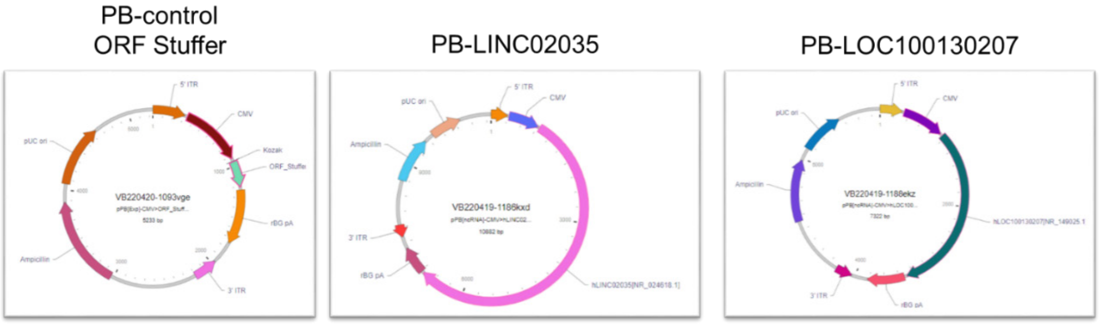

Supplementary Fig. S3. Information of the vectors used for lncRNA overexpression.

Table S5. List of primers used in the present study.

Primers	Sequence or Cat. No. of validated primers
β-ACTIN	5′-GTCCTCTCCCAAGTCCACACA-3′ (sense, NM_001101.3)<br>5′-GGGCACGAAGGCTCATCATTC-3′ (antisense)
IHH	Cat. No- P101104 V (RNA accession: NM_002181.3)<br>https://www.bioneer.co.kr/sirna-customorder.html
RUNX2	Cat. No- P229954 V (RNA accession: NM_001015051.3)<br>https://www.bioneer.co.kr/sirna-customorder.html
COL10A1	Cat. No- P261909 V (RNA accession: NM_000493.3)<br>https://www.bioneer.co.kr/sirna-customorder.html
PTGES	Cat. No- P327048 V (RNA accession: NM_004878.4)<br>https://www.bioneer.co.kr/sirna-customorder.html
BGLAP	Cat. No- P128146 V (RNA accession: NM_199173.5)<br>https://www.bioneer.co.kr/sirna-customorder.html
LINC02593	5′-CCGAGCTTCAGAGGAGAGG-3′ (sense, NR_026874.2)<br>5′-CTCCCAGATCGTGAGCTTTC-3′ (antisense)
IGFL2-AS1	5′-GGTGCCGAAGACCTGAGAC-3′ (sense, NR_135234.1)<br>5′-GGCCACTTCTTCATTCTTGTTGG-3′ (antisense)

HCG4B	5′-GTCATTCACCGGCCTAGCTC-3′ (sense, NR_001317.3)<br>5′-GAGTATTGGGACCGGAGCAC-3′ (antisense)
LINC02035	5′-GAAGCAAGCCAGAAAGCCTG-3′ (sense, NR_024618.1)<br>5′-GTGGTGCAGAGCCATGAAAG-3′ (antisense)
LOC100287175	5′-GATCAGTCTCTCTGCGCCTC-3′ (sense, NR_149003.1)<br>5′-GTGTCTCTTGGCCCTGTACC-3′ (antisense)
LMNTD2-AS1	5′-CAGGCACTCACCTACCTGAC-3′ (sense, NR_147607.1)<br>5′-CCTGGAGCAGAGGGAATACTTG-3′ (antisense)
LOC100287808	5′-ACTGGGAACTCAAGGCACAG-3′ (sense, NR_149004.1)<br>5′-AGATTCGGCTCAAAGGAGGC-3′ (antisense)
LOC100130207	5′-GCACAACAGGACAAAGGAAGG-3′ (sense, NR_149025.1)<br>5′-CAGATCGCCGTTTGCTTGG-3′ (antisense)
H19	5′-AAGAAATGGTGCTACCCAGC-3′ (sense, NR_002196.2)<br>5′-GTGCAGTGGTTGTAAAGTGC-3′ (antisense)
OBSCN-AS1	5′-CTCCTCGATGCCGTACTTG-3′ (sense, NR_073154.1)<br>5′-CCACGTTCCTATGTGAGGTG-3′ (antisense)
FOXD2-AS1	5′-CCTCCTACCAATTACACGCC-3′ (sense, NR_026878.1)<br>5′-GTACACACAGGTCGCTATGG-3′ (antisense)
LOC101927811	5′-TTAAACGAGCCCTGGATCTG-3′ (sense, NR_110119.1)<br>5′-CCCGAAACAGACTTCTGAAC-3′ (antisense)

ARHGAP5-AS1	5′-ATCGCTCGCCAACTACAGAC-3′ (sense, NR_027263.1)<br><br>5′-TGGCTTCTCGCTCCACTTTC-3′ (antisense)
A2M-AS1	5′-GGGTAGCATAGTGCCCAAGG-3′ (sense, NR_137424.1)<br><br>5′-TTTATTGGAGAGGGCCGCTG-3′ (antisense)
LOC100129034	5′-TTGCTGATGACGCAGAGGTC-3′ (sense, NR_027406.1)<br><br>5′-GGACATTATTGCCGCCGTTC-3′ (antisense)
TSC22D1-AS1	5′-GAGAGCGAGCTTCGGAAAGG-3′ (sense, NR_038381.1)<br><br>5′-TTCCTTTGAACGGCGTCGG-3′ (antisense)
LOC90246	5′-CTCCCAACCTCACCTGTGC-3′ (sense, NR_026954.1)<br><br>5′-TAGCAGGGCTTTGGATGTGC-3′ (antisense)
SNAP25-AS1	5′-AGCACCCTAACGACAACAGTC-3′ (sense, NR_040710.1)<br><br>5′-AGAACCCAGCTCCATTCATCC-3′ (antisense)
LIFR-AS1	5′-TTACAGGTGTCTGTGCGGTG-3′ (sense, NR_103554.1)<br><br>5′-TCCAGTCAATCCTTGGCATCC-3′ (antisense)
MKLN1-AS	5′-ATCTCAGGGTTTCCCACGTC-3′ (sense, NR_125364.1)<br><br>5′-ACTGTCCAGGCTTTCAGGAG-3′ (antisense)
LINC02015	5′-ACAGCCGGGAAGAAAATTGT-3′ (sense, NR_110826.1)<br><br>5′-CCAGAAGTTCATGGCAGCAG-3′ (antisense)
SOX9	Cat. No- P232240 V (RNA accession: NM_000346.3)<br><br>https://www.bioneer.co.kr/sirna-customorder.html

COL2A1	Cat. No- P298511 V (RNA accession: NM_001844.4)<br><br>https://www.bioneer.co.kr/sirna-customorder.html
SULF2	Cat. No- P102960 (RNA accession: NM_001161841.1)<br><br>https://www.bioneer.co.kr/sirna-customorder.html
FGFR4	Cat. No- P164132 (RNA accession: NM_001291980.1)<br><br>https://www.bioneer.co.kr/sirna-customorder.html
TGFBR3	Cat. No- P270889 (RNA accession: NM_001195683.1)<br><br>https://www.bioneer.co.kr/sirna-customorder.html
WNT6	Cat. No- P204423 (RNA accession: NM_006522.3)<br><br>https://www.bioneer.co.kr/sirna-customorder.html
ADAMTS5	Cat. No- P127505 (RNA accession: NM_007038.4)<br><br>https://www.bioneer.co.kr/sirna-customorder.html
MMP13	Cat. No- P221090 (RNA accession: NM_002427.3)<br><br>https://www.bioneer.co.kr/sirna-customorder.html
IGFBP3	Cat. No- P123087 (RNA accession: NM_00598.4)<br><br>https://www.bioneer.co.kr/sirna-customorder.html
COL9A2	Cat. No- P288169 (RNA accession: NM_001852.3)<br><br>https://www.bioneer.co.kr/sirna-customorder.html
DDR1	5′-GCGTCTGTCTGCGGGTAGAG-3′ (sense, NM_001202521.1)<br><br>5′-ACGGCCTCAGATAAATACATTGTCT-3′ (antisense)

RUNX3	Cat. No- P272246 (RNA accession: NM_001031680.2)<br><br>https://www.bioneer.co.kr/sirna-customorder.html
HBEGF	Cat. No- P166323 (RNA accession: NM_001945.2)<br><br>https://www.bioneer.co.kr/sirna-customorder.html
GREM1	Cat. No- P224682 (RNA accession: NM_001191322.1)<br><br>https://www.bioneer.co.kr/sirna-customorder.html
NGF	Cat. No- P319108 (RNA accession: NM_002506.2)<br><br>https://www.bioneer.co.kr/sirna-customorder.html
CXCL6	5′-GGGAAGCAAGTTTGTCTGGACC-3′ (sense, NM_002993.4)<br><br>5′-AAACTGCTCCGCTGAAGACTGG-3′ (antisense)
ADAM9	Cat. No- P104329 (RNA accession: NM_003816.2)<br><br>https://www.bioneer.co.kr/sirna-customorder.html

Table S6. siRNAs used in the current study.

siRNAs	Dose (nM)	Sequence or Cat. No of validated siRNAs
Negative control	100	Sense siRNA targeted sequence 5-CCUACGCCACCAAUUUCGU-3′<br><br>antisense siRNA targeted sequence 5-ACGAAAUUGGUGGCGUAGG-3′
IGFL2-AS1	100	RNA-GAC UCU CUU CAU AUG GAC U=tt(1-AS)<br><br>RNA-AGU CCA UAU GAA GAG AGU C=tt(1-AA)
		RNA-CUG CAA AGA AGG AAC ACU A=tt(2-AS)

HCG4B	100	RNA-UAG UGU UCC UUC UUU GCA G=tt(2-AA)
LINC02035	100	RNA-CAG GAU UGA GGA AUC UGU A=tt(3-AS)<br>RNA-UAC AGA UUC CUC AAU CCU G=tt(3-AA)
LOC100287175	100	RNA-GCA GCC UCC UCU UGU UCC U=tt(4-AS)<br>RNA-AGG AAC AAG AGG AGG CUG C=tt(4-AA)
LMNTD2-AS1	100	RNA-CUG ACU UGC UGA AAC UAC A=tt(5-AA)<br>RNA-UGU AGU UUC AGC AAG UCA G=tt(5-AA)
LOC100287808	100	RNA-CAA UUA CCU CCA GCA UAU =tt(6-AS)<br>RNA-UAU AUG CUG GAG GUA AUU G=tt(6-AA)
LINC02593	100	RNA-GAU UAC AGA GUC AUU CAU U=tt(7-AS)<br>RNA-AAU GAA UGA CUC UGU AAU C=tt(7-AA)
LOC100130207	100	RNA-CAG AAC UGC CAG CAG ACU A=tt(8-AS)<br>RNA-UAG UCU GCU GGC AGU UCU G=tt(8-AA)
RUNX2	100	siRNA ID- 860-1 (Bioneer; https://www.bioneer.co.kr/sirna-customorder.html)
